# Dissecting the Emerging Regulatory and Mechanistic Paradigms of Transcribed Conserved Non-Coding Elements in Breast Cancer

**DOI:** 10.3390/biom15050627

**Published:** 2025-04-27

**Authors:** Wenyong Zhu, Hao Huang, Qiong Li, Yu Gu, Rongxin Zhang, Huiling Shu, Yunqi Zhao, Hongde Liu, Xiao Sun

**Affiliations:** State Key Laboratory of Digital Medical Engineering, School of Biological Science and Medical Engineering, Southeast University, Nanjing 211189, China

**Keywords:** non-coding elements, transcriptional regulation, molecular mechanisms, biological function, breast cancer

## Abstract

Transcribed conserved non-coding elements (TCNEs), which are non-coding genomic elements that can regulate vital gene expression, play an unclear role in the development of severe diseases mainly associated with carcinogenesis. Currently, there are no mature tools for the identification of TCNEs. To compensate for the lack of a systematic interpretation of the functional characterization and regulatory mechanisms of TCNE spatiotemporal activities, we developed a flexible pipeline, called captureTCNE, to depict the landscape of TCNEs and applied it to our breast cancer cohort (SEU-BRCA). Meanwhile, we investigated the genome-wide characteristics of TCNEs and unraveled that TCNEs harbor enhancer-like chromatin signatures as well as participate in the transcriptional machinery to regulate essential genes or architect biological regulatory networks of breast cancer. Specifically, the TCNE transcripts could recruit RBPs, such as ENOX1 and PTBP1, which are involved in gene expression regulation, to participate in the formation of regulatory networks and the association with altered splicing patterns. In particular, the presence of a non-classical secondary structure, called RNA G-quadruplex, on TCNE transcripts contributed to the recruitment of RBPs associated with subtype-specific transcriptional processes related to the estrogen response in breast cancer. Ultimately, we also analyzed the mutational signatures of variant-containing TCNEs and discerned twenty-one genes as essential components of the regulatory mechanism of TCNEs in breast cancer. Our study provides an effective TCNE identification pipeline and insights into the regulatory mechanisms of TCNEs in breast cancer, contributing to further knowledge of TCNEs and the emergence of innovative therapeutic strategies for breast cancer.

## 1. Introduction

Breast cancer is one of the most common cancers with high morbidity and mortality rates in female patients and its incidence is gradually increasing each year worldwide [1]. Moreover, this heterogeneous disease is distinguished by its multifactorial etiology, involvement of multiple genes, and progression through distinct stages of formation [2]. Clinically, even with the same cancer stage and pathological classification, the prognosis and response to treatment still diverge due to altered molecular genetics [3]. However, the underlying genetic regulatory mechanisms involved in the pathogenesis of breast cancer remain elusive. Concurrently, the importance of genomic regulatory elements in clinical oncology is increasingly recognized, as they facilitate the prevention of carcinogenesis and the optimization of therapies for patients [4]. Therefore, identifying pivotal genomic regulatory elements in large-scale breast cancer cohorts could provide new insights into the initiation and progression mechanisms as well as clinical treatment.

Extensive molecular studies of breast cancer have revealed that non-coding regulatory elements are frequently mutated and dysregulated in different cell types and tissues, affecting the expression and function of critical genes involved in tumor initiation, progression, and response to therapy [5,6]. A category of these non-coding elements exhibits an extraordinary degree of conservation between multiple species, known as conserved non-coding elements (CNEs), which tend to cluster in the vicinity of key developmental regulatory target genes, and their disruption could contribute to carcinogenesis [7]. Our prior finding, along with those of other studies, has highlighted that transcribed non-coding elements could play essential roles in the occurrence and progression of severe diseases, particularly those associated with cancer [8,9]. Coincidentally, the majority of CNEs, designated as transcribed conserved non-coding elements (TCNEs), have been found to be transcribed in the human genome [7]. Although previous studies have suggested that conserved non-coding elements are generally transcribed, few studies have investigated this in-depth or focused on a minority of TCNEs rather than a comprehensive investigation of unidentified TCNEs in the cancer genome [10,11,12]. Furthermore, compared to other non-coding regulatory elements such as enhancers, the knowledge about TCNEs is insufficient [12], and a systematic interpretation of the functional characterization and regulatory mechanisms of TCNE spatiotemporal activities in breast cancer is still lacking.

In this study, we developed a flexible identification pipeline, captureTCNE, to depict the landscape of TCNEs, which could potentially be versatile for application in diverse diseases and datasets. To elucidate the crucial roles of TCNEs in breast cancer, we performed the identification procedure in the SEU-BRCA dataset. Then, we characterized the chromatin states, sequence and structure signatures, and associated variant profiles of TCNEs, along with the regulatory roles of the genes targeted by TCNEs. Additionally, we probed the breast cancer biology of genes potentially targeted by the identified TCNEs and the relationship between TCNEs with variants and breast cancer risk. Overall, our study deciphered the spatial transcriptional activity of TCNEs, including participating in the transcriptional machinery, regulating key genes, and shaping biological networks. Unraveling the emerging regulatory and mechanistic paradigms of TCNEs will facilitate further insights into the mechanisms of breast cancer initiation and progression, as well as promote the diagnosis and prognosis of breast cancer.

## 2. Materials and Methods

### 2.1. Preprocessing of Breast Cancer RNA-Seq Data

The raw sequence reads from 199 breast cancer samples in FASTQ format were processed in a customized RNA-seq workflow. In detail, quality control for the raw reads was performed using FastQC (version 0.11.9). Trimmomatic (version 0.39) was used to remove low-quality bases and splice sequences, with the allowance of mismatches at two positions when comparing splice sequences. Then the reads were mapped to the human reference genome (GENCODE Release 19) using STAR (version 2.7.1a). The BAM files were sorted and transformed using SAMtools (version 1.14) and bamCoverage (version 3.5.1). StringTie (version 2.2.0) was used to assemble RNA-seq alignments into potential transcripts. The quantification of gene expression levels was performed using featureCounts (version 2.0.3).

For downstream analysis, we excluded reads mapped to ribosomal RNAs, as well as chromosome Y and mitochondrial genome. Chromosome Y was excluded as our breast cancer samples were taken from females and the removal of the mitochondrial genome can reduce interfering or confusing signals [13].

### 2.2. Extraction of Known CNEs, Excluded Regions, and Independent Transcribed Regions

The known CNEs are genomic sequences defined as being more than 200 base pairs in length [14] and with conservation between human, rat, and mouse genomes [15]. We extracted the highly conserved non-coding elements of human, rat, and mouse genomes, which were detected at similarity thresholds of 80% in the genome comparison, from Ancora [16], and the ultra-conserved non-coding elements of multiple vertebrate species from UCNEbase [17]. Then, the CNEs with lengths above 200 bp were kept for downstream analysis.

The blocklist consisted of three groups: (1) multiple structural RNAs such as ribosomal RNA (rRNA), transfer RNA (tRNA), small nuclear RNA (snRNA), and small nucleolar RNA (snoRNA) from RNAcentral [18] and GENCODE [19]; (2) annotated exons, 2k bp upstream and 1k bp downstream of genes defined from GENCODE [19] and UTR regions from the UCSC [20] Table Browser Tool; and (3) genomic gap regions in the UCSC [20] hg19 assembly.

CAGE profiles of 37 human cell lines from GSE34448 [21] were selected as a pool with transcription-independent signals. To avoid the effects of noise, we excluded the lower 5% of signals in each CAGE BigWig file to screen for transcription-independent elements and confirmed that the identified elements were transcribed in at least one human sample, thus compensating for limited breast cancer data.

### 2.3. Identification Pipeline for TCNEs

**Figure 1:**
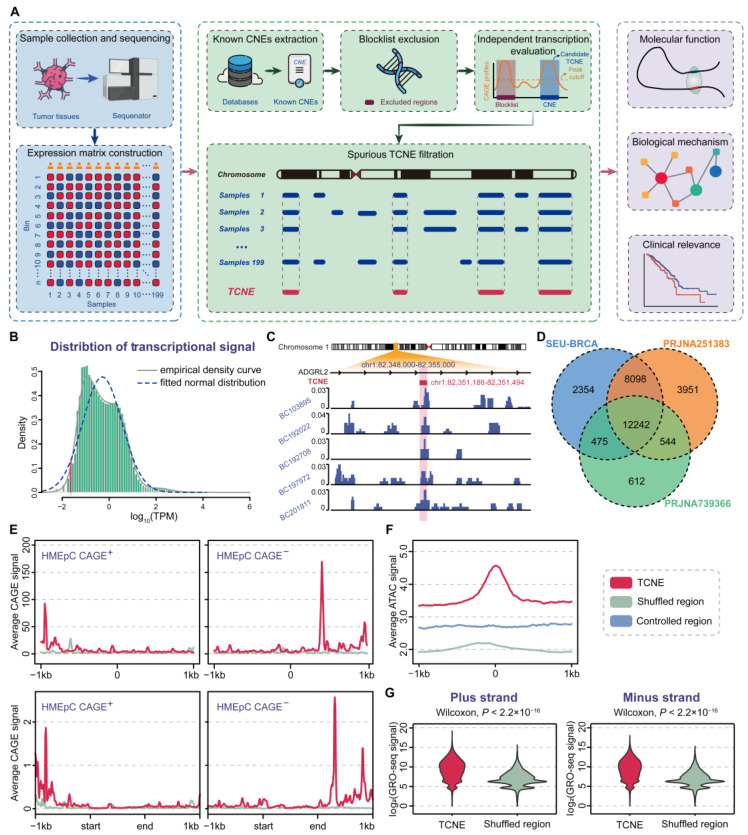
Genome-wide identification pipeline and validation of TCNEs. (**A**) The workflow of sample collection and sequencing data preprocessing, TCNE identification, and downstream analysis. (**B**) Transcriptional signal distribution of SEU-BRCA cohort, where the empirical density curve and fitted normal distribution are marked with a solid grey line and a dashed blue line, respectively, and the lower 5% cut-off is depicted in red. (**C**) RNA-seq signals of a typical TCNE in five breast cancer samples. (**D**) Comparison of TCNEs identified in three breast cancer datasets. (**E**) Comparison of the average CAGE signal between TCNEs and shuffled regions at a distance of 1000 bp each upstream and downstream (top), or after fitting the lengths to 1000 bp (bottom). (**F**) Comparison of chromatin accessibility between TCNEs and shuffled regions/controlled regions. (**G**) Comparison of GRO-seq signal between TCNEs and shuffled regions within plus strand (left) and minus strand (right).

A schematic of the TCNE identification pipeline is shown in Figure 1A. We first calculated the read density values at each genomic nucleotide position for all samples and the aggregated signals for each sample by computing the trimmed mean across all samples. Then, we scanned the aggregated signals in BedGraph format with following five filters: (1) exclude genomic regions from the blocklist mentioned above; (2) extract the candidate CNEs that were entirely within the regions with full-length transcripts of the tumor dataset; (3) ensure that retained CNEs harbor independent transcriptional signals; (4) fit RNA-seq signals to a normal distribution and define the lowest 5th percentile of transcription levels as the threshold; and (5) screen for candidate CNEs whose transcripts were detected in at least 5% of tumor samples as the final identified TCNEs.

### 2.4. Validation of the Identified TCNEs

The public breast cancer datasets were derived from NCBI under accession code PRJNA251383 and PRJNA739366. The CAGE-seq data of the human mammary epithelial cell were obtained from GEO under accession code GSM979657. To validate the chromatin accessibility of TCNEs, we generated the average signal of ATAC-seq from all breast cancer samples in the TCGA-BRCA cohort [22]. GRO-seq is the first genome-wide technique developed to probe nascent transcription genome-wide instead of steady state RNA levels. Peaks at high transcript levels in 13 different breast cancer cell lines representing the five major molecular subtypes of breast cancer detected by GRO-seq were from GEO under accession code GSE96859.

### 2.5. Construction of Shuffled Regions and Controlled Regions

Shuffled regions were chosen using shuffleBed (version 2.30.0) which excluded blocklist regions. Then, we kept features on the same chromosome of each TCNE and supplied seed 666 for the shuffling.

In the selection of controlled regions, for each intronic TCNE, all introns within 2 kb upstream and downstream of it were obtained, in which the TCNEs and the blocklist regions were excluded, and for TCNEs in intergenic regions, the controlled regions were restricted to intergenic regions of the same chromosome using the selection approach of shuffled sequences described above.

### 2.6. Characterization of TCNEs with Known Annotations

To investigate the potential roles of TCNEs in breast cancer, we characterized them using various known regulatory data in mammary tissue types or cell lines. We extracted enhancer states in any of three human mammary tissues (E027 breast myoepithelial, E028 breast vHMEC mammary epithelial, and E119 HMEC mammary epithelial) in the Roadmap Epigenomics Project [23]. Putative enhancers are marked as the E6, E7, or E12 states from the 15-state segmentation defined by five core marks using chromHMM. In addition, other known annotations consist of CAGE-defined enhancers from FANTOM5 [24], enhancers identified by histone modifications and transcriptional coactivator EP300 binding sites from ENCODE [25], and predicted enhancers across different cell lines or tissue types from EnhancerAtlas [26]. A minimum of 80% overlap with known breast cancer-associated enhancers was estimated for each TCNE prior to downstream analysis.

Meanwhile, active enhancer markers were collected from GSE85158 and ENCODE. The histone modifications H3K4me1 and H3K27ac from 13 breast cancer cell lines under accession code GSE85158 were integrated and EP300 (ENCSR000BTR) as well as CTCF (ENCSR000DWH) signals of breast cancer cell line MCF-7 from ENCODE were selected.

### 2.7. Clinical Analysis of TCNEs with Enhancer Signatures

The quantification of typical enhancers in TCGA-BRCA was derived from TCeA [27] and clinical data for TCGA-BRCA were collected from cBioPortal [28]. In Cox regression analysis, we captured TCNEs overlaid with typical enhancers using intersectBed (version 2.30.0) with parameters ‘-f 0.5 -r’. Based on the median expression levels in TCGA-BRCA samples, TCNEs were categorized into two groups to study the clinical relevance of TCNEs.

### 2.8. Motif Discovery and Enrichment Analysis

For motif discovery for intronic TCNEs, we performed a differential enrichment analysis using MEME (version 5.5.3) to retrieve motifs specific to the first intronic TCNEs, treating the other intronic TCNEs as control sequences. Then, for the most significantly enriched motifs, average likelihood ratio scores for all feasible binding events were calculated using AMA (version 5.5.3), and the associated GO terms were scanned using GOMo (version 5.5.3).

The plus and minus strand information for TCNEs was detected according to the transcripts assembled by StringTie (version 2.2.0). Motif discovery analysis was performed on sequences of TCNE transcripts using MEME-ChIP (version 5.5.3) with the parameter ‘-ccut = 0 -seed = 666’. In addition, the minimum and maximum lengths of motifs in the parameter settings were 10 and 30, respectively, based on the length of RNA G-quadruplex (rG4) in the G4Atlas database (Supplementary Figure S1A) and the input limits of the tools. The motif enrichment analysis was conducted using AME (version 5.5.3) to identify known RBP motifs enriched in the sequences of TCNE transcripts. The optimal enrichment of the motifs was performed with the one-tailed Fisher’s exact test, the p value was adjusted with the Bonferroni correction, and the seed was set to 666. All of these RBPs enriched by TCNE transcripts were used as input to the QUADRatlas database [29] to search for rG4-binding proteins.

rG4 motifs were retrieved using pqsfinder (version 2.14.1) to filter rG4-containing TCNE transcripts. Based on a previous study [30], it was found that pqsfinder outperformed all of the other algorithms when setting a score threshold of 25.

The ensemble free energy of TCNEs with and without rG4 motifs was calculated separately using RNAfold (version 2.6.4) with parameters ‘-p0 --noPS --noDP –gquad’.

### 2.9. Integration of Gene Expression Data in Breast Cancer Tissues and NATs

We used TCGAbiolinks (version 2.26.0) to obtain the gene expression profile (TPM) of breast cancer patients from TCGA, which contains 1099 breast cancer tissues and 112 normal tissues adjacent to the tumor (NATs). Meanwhile, we extracted the ‘gene_tpm_2017-06-05_v8_breast_mammary_tissue.gct.gz’ file from GTEx, which contained 459 normal breast/mammary tissue samples. Then, we merged the gene expression data of TCGA and GTEx and obtained adjusted TPM after removing the batch effect using combat-seq from sva (version 3.46.0, Supplementary Figure S1B,C).

### 2.10. Prediction of Target Genes Associated with TCNEs

To predict the links between TCNEs and target genes, we restricted the range of interaction length to 500 k bp to avoid spurious predictions, based on the fact that more than 75% of three-dimensional promoter-based interactions occur within a distance of 500 k bp [31]. Copy number aberrations (CNAs) were particularly important in these links as a strong driver for spurious TCNE-to-gene correlation in breast cancer. These false-positive links were eliminated if the log2 scale of the segment mean value, which was extracted from PanCancer Atlas [28], was above 1.5. For each candidate TCNE-to-gene link, we individually applied Spearman’s rank correlation coefficient and then selected the eligible links (those with BH-adjusted p<0.01 and Spearman’s rank correlation coefficient $\left|\rho \right|>0.8$ were considered as the links of TCNEs and predicted genes).

### 2.11. Pathway, GO, and GWAS Enrichment Analysis

For pathway and GO enrichment analysis of genes, clusterProfiler (version 4.6.2) was used, while msigdbr (version 7.5.1) was employed to retrieve known gene sets from the MSigDB database. GWAS disease/trait enrichment analysis was fulfilled using Enrichr (last update available on 8 June 2023). For pathway and GO enrichment analysis of genomic regions, rGREAT (version 2.0.2) was utilized to study the functions of the regulatory elements.

### 2.12. Construction of Gene Regulatory Network

To explore important nodes/hubs in an interactome network comprising TCNE-targeted genes, STRING (version 12.0) was applied to search for gene regulatory networks of all target genes. Cytoscape (version 3.10.1) was utilized to discover featured nodes, with a topological algorithm, namely Maximal Clique Centrality (MCC), where ‘Hubba nodes’ were set to the top six.

### 2.13. Variant Annotation and Analysis on TCNEs

Consensus SNV/Indel related to breast cancer was retrieved from ICGC PCAWG [32] with 787,272 entries of variant sites. Then, we used intersectBed (version 2.30.0) to find the variant sites located on TCNEs. Meanwhile, to perform the permutation test, we also utilized shuffleBed (version 2.30.0) to choose random regions entirely contained in non-coding transcripts assembled by StringTie (version 2.2.0). The expression quantitative trait loci (eQTL) data were derived from breast/mammary tissue-related information in the ‘GTEx_Analysis_v8_eQTL.tar’. Mutational signatures (version 2, March 2015) of the above variants have been identified and compared with those available in COSMIC via the R package deconstructSigs (version 1.8.0). Additionally, to provide more accurate analyses and to reduce the interference of false positives, of 883 variant-containing TCNE-associated genes, 332 genes associated with at least two TCNEs with observed variants were shortlisted for enrichment analysis.

### 2.14. Cell Culture and Quantitative PCR

All cell lines used in this research were acquired from the cell resource center of Shanghai Institutes for Biological Sciences, Chinese Academy of Sciences. The human breast cancer cell lines MCF-7, T-47D, BT-474, and MDA-MB-231 were used in this study. In addition to the tumorigenic cell lines, the non-tumorigenic human mammary epithelial cell line MCF-12A was also included in the experiments. BT-474 was cultured in RPMI-1640 (GIBCO, Waltham, MA, USA). MDA-MB-231 was cultured in Leibovitz’s L-15 (GIBCO). MCF-7, T-47D, and MCF-12A were cultured in Dulbecco’s Modified Eagle Medium (DMEM, Gibco). All media were supplemented with 10% fetal bovine serum (HyClone, Logan, Utah), 100 units/mL penicillin (Thermo Fisher, Waltham, MA, USA), and 100 μg/mL streptomycin (Thermo Fisher). Cells were incubated at 37 °C in a humidified incubator containing 5% CO_2_. Total RNA was isolated from the cultured cells using TRIzol™ (Invitrogen, Carlsbad, CA, USA) according to the manufacturer’s protocol. The complementary DNA (cDNA) was generated using the PrimeScriptTM RT reagent kit with gDNA Eraser (Takara, Kusatsu, Japan). The expression of target genes from cDNA was detected with quantitative PCR (qPCR) on an ABI StepOne Plus (Applied Biosystems, Waltham, MA, USA) by using the RR820Q TB Green^®^ Premix Ex Taq™ II (Tli RNaseH Plus, Takara). Each sample was detected in four technical replicates. The relative mRNA transcript level was calculated as 2^−ΔCt^, in which, ΔCt = Ct_target_ − Ct_GADPH_. 2^−ΔCt^ was also defined as relative quantity (RQ). The specificity of all qPCR primers was verified using melting curve analysis (Supplementary Table S1).

### 2.15. Statistical Analysis

Continuous variables were compared using the Wilcoxon signed-rank test and Spearman’s rank correlation coefficient, and categorical variables were compared using the permutation test or Fisher’s exact test. The overall survival probabilities were estimated using the Kaplan–Meier method and compared using the log-rank test. The statistical significance threshold was set at p<0.05. The Bonferroni–Holm (BH) procedure was used in multiple hypothesis testing to reduce false positive rates. Statistical analyses were performed with R (version 4.2.3).

## 3. Results

### 3.1. Genome-Wide Identification and Validation of TCNEs in Breast Cancer

To systematically and comprehensively characterize the TCNE landscape in breast cancer, a sophisticated pipeline with a strict five-step filter, named captureTCNE, was developed to identify TCNEs (Figure 1A). Here, we required TCNEs for each sample to fulfill the following criteria: (1) to be without overlap with regions that encode structural RNAs, exons, and genomic gap regions; (2) to be the complete sequences that were transcribed into RNA molecules; and (3) to be transcribed independently from overlapping genes. We then determined the candidate TCNEs that (4) were above the lowest fifth percentile of transcription levels and (5) detected in at least 5% of tumor samples.

We applied catpureTCNE to the SEU-BRCA cohort, including the ribosomal RNA-depleted RNA sequencing of 199 breast cancer patients, as previously described in detail [8]. Consequently, 23,169 TCNEs were discovered (Figure 1B), and it was confirmed that TCNEs were indeed in non-coding regions with independent transcriptional signals (Figure 1C). Concurrently, we utilized multiple external datasets to substantiate the rationality and accuracy of our identification pipeline. It was found that 20,815 (89.84%) of the TCNEs identified in the SEU-BRCA cohort could also be detected in other breast cancer datasets (Figure 1D), suggesting the robustness of captureTCNE. To further verify the identified TCNEs were transcription-independent instead of the result of coding gene expression, independent transcriptional signals between TCNEs and shuffled regions of known CNEs without discernable transcripts were compared and revealed that the identified TCNEs were transcription-independent in the plus strand and minus strand, respectively ($p=7.48\times 10^{-13}$ and $p=1.53\times 10^{-18}$, Wilcoxon signed-rank tests, Figure 1E). Then, we extracted ATAC-seq data from all breast cancer samples of the TCGA-BRCA cohort [22] and contrasted chromatin accessibility between TCNEs and shuffled regions (or controlled regions), respectively. As expected, TCNEs were enriched with a higher level of chromatin accessibility, indicating that TCNEs are in transcriptionally activated states ($p<2.2\times 10^{-16}$, Wilcoxon signed-rank test, Figure 1F). Ultimately, in a public breast cancer cohort (GSE96859) where nascent RNAs were profiled using GRO-seq, over 21,349 (92.14%) TCNEs were derived, and it was observed that the transcription levels of the identified TCNEs were significantly higher than those of shuffled regions ($p<2.2\times 10^{-16}$, Wilcoxon signed-rank test, Figure 1G). Considering all of the aforementioned evidence, our pipeline could reasonably and accurately identify transcription-independent TCNEs in breast cancer and be prospectively applicable to other cancer types.

### 3.2. Characterization of TCNEs Reveals Their Functions in the Regulation of Host Genes

**Figure 2:**
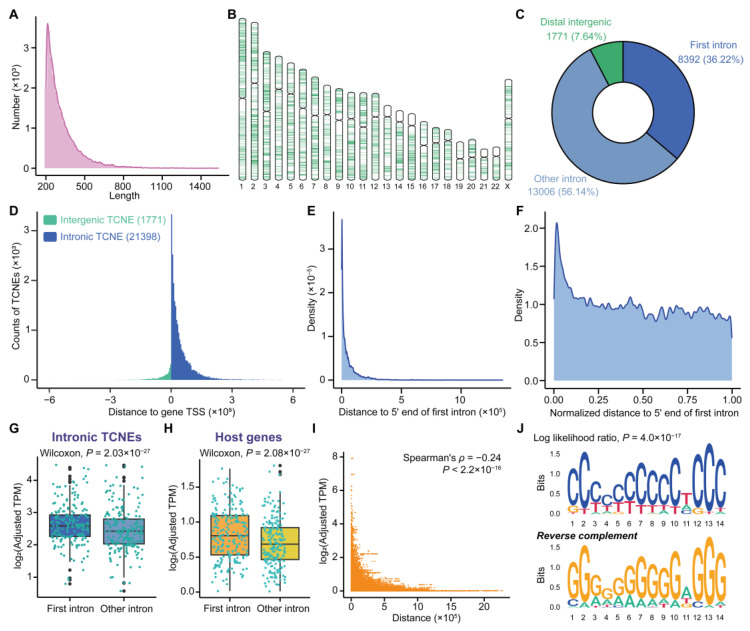
The landscape of TCNEs in breast cancer. (**A**) Size distribution of TCNEs. (**B**) Genome-wide overview of the location of TCNEs identified in our breast cancer cohort. (**C**) Genomic location distribution of TCNEs. (**D**) Distribution of distances from the intronic TCNEs to the host gene TSS (blue) or the intergenic TCNEs to the nearest gene TSS (green). (**E**) Physical distance, or (**F**) normalized distance, from the middle points of TCNEs located in the first intron to the host gene TSSs. Comparison of transcription levels of (**G**) intronic TCNEs, or (**H**) host genes, located in the first intron and other introns. (**I**) The correlation between the expression levels of host genes and the distance from TCNEs to host gene TSSs. (**J**) A significantly enriched motif for the first intronic TCNEs against the other intronic TCNEs.

Given the accuracy and reliability of the TCNEs identified in breast cancer, we proceeded to characterize TCNEs in silico to ascertain their impacts on host genes. It is noteworthy that host genes were defined as genes containing intronic TCNEs, and they were potential targets of TCNEs. Of the 23,169 TCNEs identified in the SEU-BRCA cohort, the sizes of 22,856 (98.56%) TCNEs were clustered in the range of 200–800 bp (Figure 2A) and the distribution of TCNEs was not uniform across all chromosomes (Figure 2B). Interestingly, the vast majority of TCNEs (21,398, accounting for 92.36%) were situated in intronic regions, of which 8392 (36.22%) TCNEs were located in the first intron of the host genes (Figure 2C). Meanwhile, the TCNEs were distributed in a manner that is adjacent to the transcription start sites (TSSs) of the host genes (Figure 2D), and the intronic TCNEs also tended to bias toward the 5′ end of the host gene body (Figure 2E,F).

Previous studies have shown that the first introns are significantly enriched for various genomic regulatory elements, which could affect the transcription level of host genes [33,34]. In light of the previous observation that TCNEs were enriched in the first introns, it was necessary to verify whether TCNEs affected the expression of their host genes. Upon comparing the transcriptional profiles of first intronic TCNEs with that of other intronic TCNEs, it turned out that the transcription levels of first intronic TCNEs were significantly higher ($p=2.03\times 10^{-27}$, Wilcoxon signed-rank test, Figure 2G), and the host genes of the first intronic TCNEs also exhibited higher transcription levels ($p=2.08\times 10^{-27}$, Wilcoxon signed-rank test, Figure 2H). In addition, the transcriptional signals of the first intronic TCNEs were higher than other intronic TCNEs (Supplementary Figure S2A), and the expression level of the first intronic TCNEs was significantly correlated with that of the host genes (Supplementary Figure S2B–I). In light of the foregoing comparative outcomes, the correlation between the distance of the intronic TCNEs to host gene TSSs and the expression levels of the host genes were also evaluated. It was uncovered that the proximity of intronic TCNEs to the host gene TSSs leads to higher expression levels of the host genes (Figure 2I), as well as higher transcriptional signals (Supplementary Figure S2J,K). In contrast to the other intronic TCNEs, a significantly enriched motif was discovered on the first intronic TCNEs (Figure 2J), and the most significant specific biological role of this motif was designated as ‘positive regulation of transcription from RNA polymerase II promoter’ (GO:0045944, BH-adjusted $p=5.38\times 10^{-6}$) using the GOMo tool. Moreover, the identified TCNEs exhibited significant differences in transcription levels in a public cohort (PRJNA739366) containing both NAT and breast cancer ($p=4.96\times 10^{-15}$, Wilcoxon signed-rank test, Supplementary Figure S2L). Collectively, a negative correlation was found between the distance from intronic TCNEs to host gene TSSs and the expression levels of host genes, as well as transcriptional signals, suggesting that the TCNEs might be involved in the transcription machinery to regulate the host genes.

### 3.3. A Subset of TCNEs Serves as Putative Enhancers Active in Breast Cancer

In the intricate landscape of cancer genomes, CNEs act as important drivers and potentially function as enhancers to maintain sites for transcription factor occupancy [14]. The identified TCNEs harbored an open chromatin conformation and contained the higher H3K27ac signals. To further dissect the functions of TCNEs, we inspected whether TCNEs were defined as enhancers and then investigated whether their transcripts functioned as enhancer RNAs (eRNAs), facilitating the dynamic interaction between enhancers and promoters.

**Figure 3:**
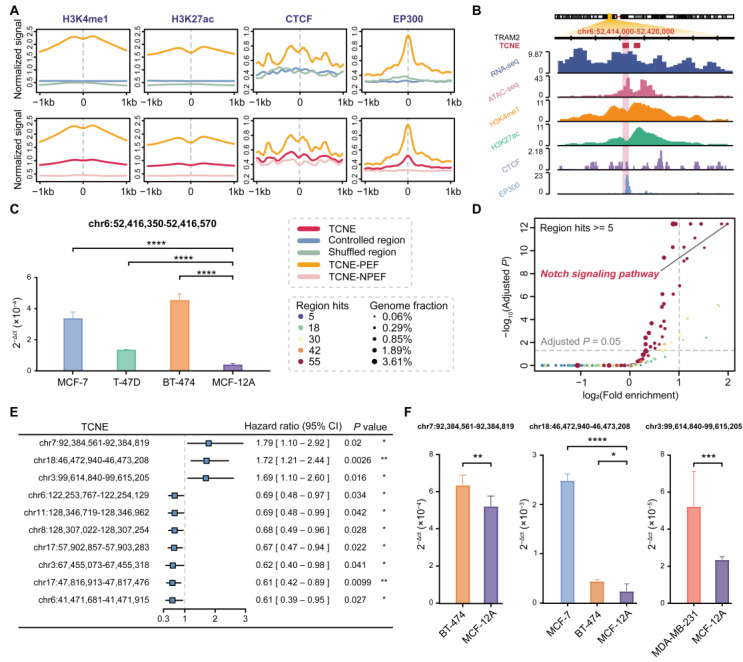
Characterization of TCNEs based on putative enhancer features. (**A**) Comparison of histone modification marks (H3K4me1 and H3K27ac), CTCF signal, and EP300 signal between TCNEs (red) and controlled regions (blue)/shuffled regions (green) or TCNEs with putative enhancer features (orange) and TCNEs without putative enhancer features (pink) at 1000 bp each upstream and downstream. TCNE-PEF: TCNEs with putative enhancer features. TCNE-NPEF: TCNEs with putative enhancer features not previously identified in publications. (**B**) A typical example of TCNEs located within TRAM2 enriched for multiple signals. (**C**) qPCR analysis of the TRAM2-TCNE in four cell lines. (**D**) Functional annotation of TCNEs with putative enhancer features. (**E**) Forest plot of ten TCNEs as independent clinical prognostic factors. (**F**) qPCR analysis of these three TCNEs, which were considered as risk factors, in multiple cell lines. * *p* < 0.05, ** *p* < 0.01, *** *p* < 0.001, and **** *p* < 0.0001.

By overlapping the genomic locations of TCNEs with known putative enhancers, we discovered that the TCNEs identified in the SEU-BRCA cohort were partially supported by epigenetic and genetic features of active enhancers. These features incorporated characteristic histone modifications (such as H3K4me1 and H3K27ac), CTCF binding, and transcriptional coactivator EP300 binding that were derived from the Roadmap Epigenomics Project [23] and ENCODE [25]. Among all identified TCNEs, 7241 (31.25%) TCNEs were identified as putative enhancers active in breast cancer with at least 80% overlap. Validation by comparing TCNEs with randomly shuffled regions revealed that TCNEs could be obtained as more putative enhancers than expected by chance alone ($p<2.2\times 10^{-16}$, permutation test). To comprehensively explore the chromatin status of the identified TCNEs with putative enhancer features, we calculated the average chromatin modification signals and discovered that these TCNEs have distinctly higher active enhancer markers compared to shuffled regions as well as controlled regions (Figure 3A). For instance, TRAM2-TCNE (chr6:52,416,350–52,416,570) was located in the first intron of the human gene TRAM2 and harbors high levels of active enhancer signals (Figure 3B). Meanwhile, TRAM2-TCNE was detected to be significantly overexpressed in three breast cancer cell lines, MCF-7, T-47D, and BT-474, compared to a non-tumorigenic mammary epithelial cell line, MCF-12A (Figure 3C). Previous studies have also shown that enhancer-driven TRAM2 activation is correlated with the expression and activation of the co-transcriptional factor YAP in nearly all tumor types, facilitating specific programs for cell proliferation, migration, and invasion during tumorigenesis [35,36], and high TRAM2 expression correlates with poor patient survival probability [35].

Furthermore, we unraveled the biological function of the TCNEs with active enhancer features and showed that these TCNEs were significantly associated with principal pathways involved in the initiation, invasion, and migration of breast cancer, such as the Notch signaling pathway, Pathways in cancer, TGF-beta signaling pathway, Wnt signaling pathway, MAPK signaling pathway, Hedgehog signaling pathway, and ErbB signaling pathway (Figure 3D). It was reasonable to infer that the transcripts of TCNEs with putative enhancer features also play an essential role in the regulatory networks that control gene expression in breast cancer, providing novel opportunities for targeted cancer therapies. Then, following the analysis of the transcription levels of typical enhancers (eRNA expression) that overlapped with TCNEs and clinical information from breast cancer samples (Methods), ten of these TCNEs were independent clinical factors (Supplementary Figure S3). As shown in Figure 3E, three of these TCNEs were defined as risk factors. Furthermore, these three TCNEs were also revealed to be expressed at significantly higher levels in breast cancer cell lines than in a non-tumorigenic mammary epithelial cell line, and could act as valuable predictors of disease risk and facilitate diagnostic decisions (Figure 3F).

### 3.4. Sequence and Structure Signatures of TCNE Transcripts Relevant to Breast Carcinogenesis

**Figure 4:**
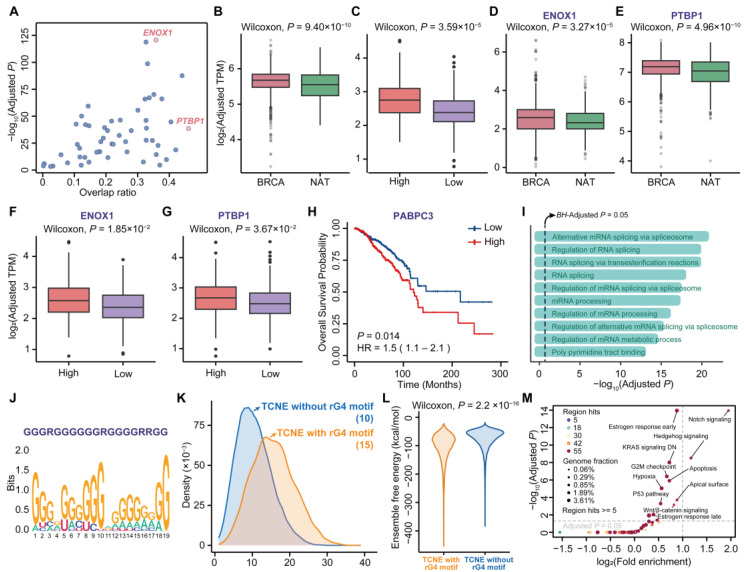
Sequence patterns and structural features of TCNEs in breast cancer. (**A**) Distribution of TCNEs enriched with the corresponding quantity of RBP motifs; overlap ratio represents the percentage of sequences enriched with motifs. Differences in average expression levels of (**B**) twenty RBPs, (**D**) ENOX1, and (**E**) PTBP1, between breast cancer (BRCA) and normal tissues adjacent to the tumor (NAT) from TCGA and GTEx. Differences in average expression levels of related TCNEs between high and low expression groups of (**C**) twenty RBPs, (**F**) ENOX1, and (**G**) PTBP1. (**H**) Kaplan–Meier survival plots of PABPC3. (**I**) GO enrichment analysis for twenty RBPs. (**J**) RNA G-quadruplex motif discovered in transcripts of TCNEs. (**K**) Comparison of RBP-enriched counts, and (**L**) comparison of ensemble free energy between TCNE with rG4 motif and TCNE without rG4 motif. (**M**) Function annotation of TCNEs with rG4 motifs.

Previous experimental evidence suggests that the transcripts of conserved regulatory elements play regulatory roles, but little is known about the regulatory motifs contained within them [37]. To elucidate the potential regulatory roles of TCNE transcripts, RNA-binding protein (RBP) motif enrichment analysis was performed (Supplementary Table S2 and Figure 4A) and most TCNE transcripts were enriched for multiple RBP motifs (Supplementary Figure S4A). In addition, it was found that 33 RBPs exhibit a significant positive correlation in expression levels with their paired TCNE transcripts (p<0.05, Spearman’s rank correlation coefficient). In addition, twenty of these RBPs had significantly higher expression levels in breast cancer compared to NAT in TCGA and GTEx datasets (Figure 4B) and a high expression of RBPs in breast cancer also correlated with a high expression of TCNEs (Figure 4C). Among all enriched RBPs, ENOX1 was obtained with the most significant p value (Bonferroni adjusted $p=1.89\times 10^{-121}$, Fisher’s exact test), and PTBP1 was enriched in the maximum quantity of TCNEs (10,677, 46.08%, Bonferroni adjusted $p=3.10\times 10^{-39}$, Fisher’s exact test, Figure 4A). Both ENOX1 and PTBP1 evinced the same trend as the ensemble of these RBPs, that is, their expression levels are positively correlated with those of the associated TCNEs as well as breast cancer development (Figure 4D–G). Simultaneously, one of the twenty RBPs, PABPC3, was significantly associated with poorer survival (Figure 4H) and these RBPs were significantly associated with the splicing process (Figure 4I), contributing to the enhancement of host gene expression [38]. Collectively, these findings demonstrated that there were synergistic effects between the identified TCNE transcripts and RBPs, and it can be postulated that these TCNE transcripts could potentially recruit RBPs and influence the chromatin state, making it more accessible for transcription machinery and thus promoting gene expression [39,40].

The RNA linear chains typically fold into secondary structures to ensure molecular stability and perform specific biological functions [41]. To further investigate the secondary structures incorporated on TCNE transcripts, we performed comprehensive motif discovery using the MEME Suite [42] and uncovered that TCNE transcripts were significantly enriched for an rG4 motif, which is a non-classical secondary structure formed by guanine-rich sequence (Figure 4J). Then, we searched for rG4 motifs within the sequence of each TCNE transcript using pqsfinder with the optimal thresholds (see Methods for details) and found that 5437 (23.47%) TCNE transcripts harbored rG4 motifs ($p<2.2\times 10^{-16}$, permutation test). For example, one of the rG4-containing TCNE transcripts with the highest pqsfinder score (chr10:76,586,543–76,586,748, Supplementary Figure S4B) was derived in the intron of KAT6B. KAT6B has been found to play an important role in ERα regulation and contribute to breast cancer cell proliferation [43]. Although the transcription levels of rG4-containing TCNEs were significantly higher ($p=2.25\times 10^{-19}$, Wilcoxon signed-rank test) than other TCNEs, there were no statistically significant differences in the average expression levels of the host genes between the two groups (p=0.63, Wilcoxon signed-rank test). Nevertheless, it was discovered that rG4-containing TCNE transcripts could recruit more RBPs (Figure 4K), contributing to significant regulatory roles in gene expression and cellular responses [44,45]. For instance, one rG4-binding protein, HNRNPL, recruited by rG4-containing TCNE transcripts, could increase the stability of transcripts and subsequently influence gene expression, contributing to breast cancer metastasis [46]. In addition, rG4-containing TCNE transcripts harbored significantly lower free energy (Figure 4L, $p<2.2\times 10^{-16}$, Wilcoxon signed-rank test), indicative of the overall stability of the RNA structure ensembles. To explore the biological function of the rG4-containing TCNEs, we applied GREAT analysis on genomic regions of these TCNEs. As illustrated in Figure 4M, we concluded that rG4-containing TCNEs were not only involved in pathways that could promote tumor initiation and progression (such as Notch signaling, Hedgehog signaling, KRAS signaling DN, P53 pathway, and Wnt/β-catenin signaling), but also associated with estrogen response in breast cancer. In summary, a subset of the TCNE transcripts incorporated the rG4 motif, resulting in structural stability and accessibility to RBPs, and were significantly associated with breast cancer initiation and progression.

### 3.5. TCNEs and Their Targeted Genes Construct the Cancer Biological Regulatory Networks

**Figure 5:**
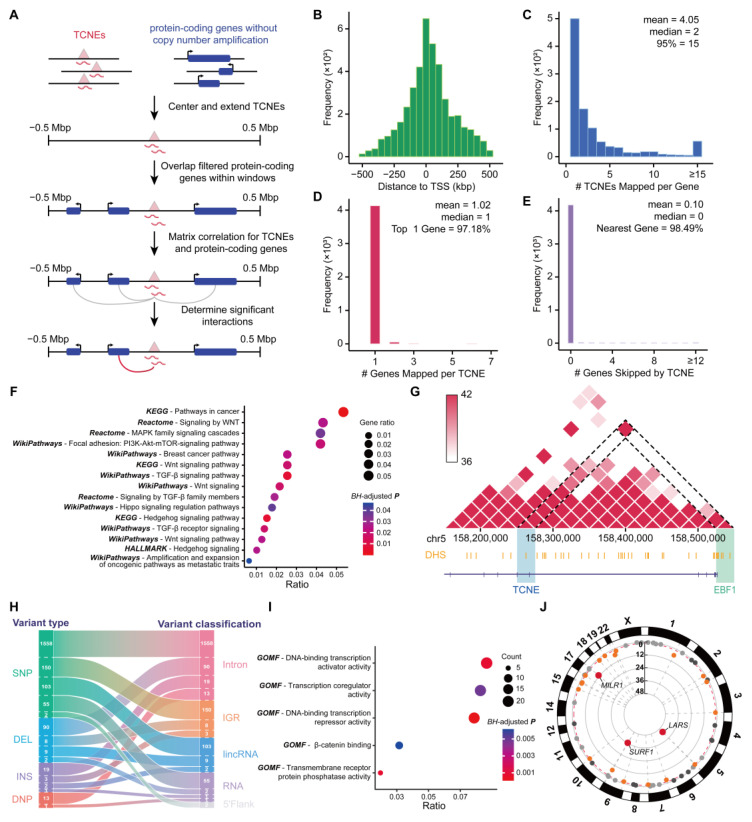
Assignment of TCNEs to associated genes in breast cancer. (**A**) Schematic of the in silico approach used to link TCNEs to genes via correlation of expression levels between TCNEs and genes. (**B**) Distribution of the distance of each TCNE to the transcription start site of the linked gene. (**C**) Distribution of the number of TCNEs mapped per gene. (**D**) Distribution of the number of genes mapped per TCNE. (**E**) Distribution of the number of genes “skipped” by a TCNE to reach its predicted linked gene. (**F**) Dot plot for pathway enrichment analysis of linked genes. (**G**) Heatmap of the interaction between the chromatin region of TCNE-EBF1 (chr5:158,122,927–158,526,769) and the EBF1 gene promoter. (**H**) Sankey plot between variant type and variant classification of variants located in TCNEs. (**I**) GO enrichment analysis, and (**J**) eQTL analysis, of the genes associated with at least two TCNEs with observed variants.

Since it has previously been demonstrated that TCNEs could regulate gene expression, we further assigned putative target genes for each TCNE, to elucidate the biological regulatory network in which the TCNE targeted genes were involved. Identifying the target genes of these regulatory elements can be valuable in pinpointing potential driver genes and signaling pathways that are critical for cancer initiation and progression [47,48]. To obtain TCNE-to-gene links, a pipeline for linkages between TCNEs and target genes was established (Figure 5A), and the workflow involved the extraction of genes that were significantly associated with the expression of each TCNE within an intersection range of 500k bp, with the exclusion of CNAs. CNAs are particularly important as a strong driver for spurious TCNE-to-gene links [22] and these false-positive links were removed through the use of TCGA DNA copy number segments data.

The pipeline identified 4251 pairs of TCNE-to-gene links (BH-adjusted p<0.01 and Spearman’s rank correlation coefficient $\left|\rho \right|>0.8$). Notably, the links between TCNEs and target genes were positively correlated and a total of 4187 TCNEs and 1049 predicted genes were included in these TCNE-to-gene links. The distance distribution of the predicted TCNE-to-gene links showed that TCNEs tended to congregate around the TSSs of target genes (Figure 5B). A single gene could be co-regulated by multiple TCNEs (Figure 5C), and yet 97.18% TCNEs involved in TCNE-to-gene links were expected to target only one linked gene (Figure 5D). Of the 4187 (98.49%) TCNE-to-gene links, the target genes were either overlapping with or closest to TCNEs (Figure 5E). To elucidate the regulatory roles of these genes targeted by TCNEs in breast cancer, we conducted pathway enrichment analysis on these associated genes. Among all 85 significantly enriched pathways (BH-adjusted p<0.05), 65 pathways had been demonstrated that were relevant to breast cancer biology (Supplementary Figure S5A and Table S3 attached with references). It was noteworthy that these significantly enriched pathways included the pathways that are well known to be significantly associated with cancer, particularly breast cancer, i.e., Pathways in cancer (BH-adjusted $p=4.42\times 10^{-4}$) and the breast cancer pathway (BH-adjusted $p=2.37\times 10^{-2}$). Fifteen pathways were highlighted as being significantly associated with breast cancer and widely acknowledged in previous studies [49] (Figure 5F). In GO enrichment analysis, it was also revealed that these target genes were significantly associated with the positive regulation of transcription, cell differentiation, and migration. For pathways, the Wnt signaling pathway, which was involved in the renewal, cell proliferation, and differentiation of cancer stem cells, resulting in carcinogenesis and therapy resistance, was also obtained (Supplementary Figure S5B and Supplementary Table S4). Moreover, these TCNE target genes were associated with an essential biological regulatory network in breast cancer, which was assigned to the PI3K-Akt signaling pathway after matching with known genetic pathways (Supplementary Figure S5C). Notably, the PI3K-Akt signaling pathway plays a significant role in breast cancer pathogenesis and serves as an important target for therapeutic intervention to overcome resistance and improve patient outcomes [50].

In addition, we evaluated the accuracy of TCNE-to-gene links using published Hi-C datasets of breast cancer tissues and cell lines, and the outcome suggested that 3916 (92.12%) links were confirmed with chromatin loops, implying that TCNEs and the promoter of target genes (2000 bp upstream of TSSs) were in spatial proximity, suggesting that TCNEs could coordinate the temporal and spatial expression of genes in biological regulatory networks. For instance, as shown in Figure 5G, the chromatin region of TCNE-EBF1 (chr5:158,257,670–158,257,898) and that of the EBF1 gene promoter showed a strong interaction, and it had been demonstrated that EBF1 is highly expressed in triple-negative breast cancer cells, and the knockdown of EBF1 can block tumor growth and invasion [51].

### 3.6. Variant-Containing TCNEs Induced Alterations of Gene Expression Related to Breast Cancer

Non-coding regulatory elements play a pivotal role in regulating gene expression and variants within these elements can significantly influence gene activity, thereby contributing to the complexity of human traits and the susceptibility to various diseases [52]. To investigate the biological mechanisms of TCNEs with complex functional variants in altering breast cancer biological networks, we highlighted the genes targeted by variant-containing TCNEs and the association between these genes and breast cancer risk. Firstly, we retrieved the SNV/Indel sites related to breast cancer from ICGC PCAWG [32] and discovered 2016 known variant sites covered 1894 TCNEs, which was significantly higher than expected by chance alone ($p<2.2\times 10^{-16}$, permutation test). Most TCNEs harbored a single variant site (Supplementary Figure S5D), and the variants located in TCNEs were mainly intronic single nucleotide polymorphisms (SNPs, Figure 5H and Supplementary Figure S5E,F).

Additionally, 883 genes supported by at least one variant-containing TCNE were subjected to enrichment analysis, and the breast cancer pathway was significantly enriched with BH-adjusted $p=1.62\times 10^{-17}$ and odds ratio=4.04 in GWAS Catalog. Then, the enrichment analysis for variant-containing TCNE-associated genes also revealed that these genes were strongly associated with transcriptional and post-transcriptional regulation, as well as the pathways in cancer, specifically the β-catenin binding pathway (Figure 5I). Additionally, twenty-one genes were observed to be targeted by variant-containing TCNEs with eQTL data from GTEx and the top three genes (LARS, SURF1, and MILR1) were significantly related to specific genetic variant loci of breast cancer ($Storey’s\;Q<1\times 10^{-8}$, Figure 5J). Specifically, the variant of TCNE associated with LARS occurred within the LARS gene body and it has been demonstrated that the monoallelic genetic deletion of LASR in the mammary gland can promote tumor formation and proliferation [53].

Furthermore, to investigate the possible etiology of the mutational processes underlying the identified TCNEs, the mutational signatures in COSMIC were evaluated. It was discovered that five mutational signatures were associated with variants retained on TCNEs, including signature 1, signature 2, signature 3, signature 5, and signature 13 (Supplementary Figure S5G,H), which have been proven to be associated with the risk of breast cancer [54]. In particular, signature 1 and signature 5 arise in all cancer types and most cancer samples, signature 3 is strongly associated with the epigenetic silencing of RAD51C and BRCA1 by promoter methylation in breast cancer, and signature 2 and signature 13 are associated with the activities of the APOBEC machinery, contributing to susceptibility to breast cancer [54].

## 4. Discussion

CNEs are a category of non-coding elements with exceptional conservation across species and typically regulate gene expression by serving as binding sites for transcription factors and other regulatory proteins, potentially contributing to carcinogenesis. As a type of CNE capable of transcription, TCNEs play essential roles in the onset of severe diseases mainly associated with carcinogenesis. Since only a few TCNEs have been characterized in the human genome [12], and the tremendous attention in the field has been concentrated on the evolutionary implications, the genome-wide identification and characterization of TCNEs are still lacking, especially in diseases like cancer. Our study was motivated by the lack of a systematic view to interpret the functions and regulatory mechanisms of TCNE spatiotemporal activities in the human cancer genome [7,15,52].

In this study, we developed a flexible pipeline for the genome-wide TCNE identification and applied it to the SEU-BRCA cohort consisting of ribosomal RNA-depleted RNA-seq, which could potentially be versatile for application in other cancer types or single-cell RNA sequencing (scRNA-seq) datasets. This identification pipeline is persuasive and user-friendly, and comprehensive instructions and a demo are available in the online repository. Here, we examined the transcription levels of the TCNEs identified in the SEU-BRCA cohort and discovered that nearly ninety percent of the TCNEs were also transcribed in other public cohorts of breast cancer. We utilized CAGE-seq data of breast cancer cell lines combined with ATAC-seq data collected from TCGA and revealed that the TCNEs were truly transcribed instead of transcriptional noise. Furthermore, we ascertained that more than ninety percent of the identified TCNEs were also detected by GRO-seq, which can derive the location and orientation of all actively transcribing RNA polymerases across the genome [55]. All of these experiments affirmed the reliability of our methodology, which could potentially be versatile for application in diverse cancer types and datasets.

**Figure 6:**
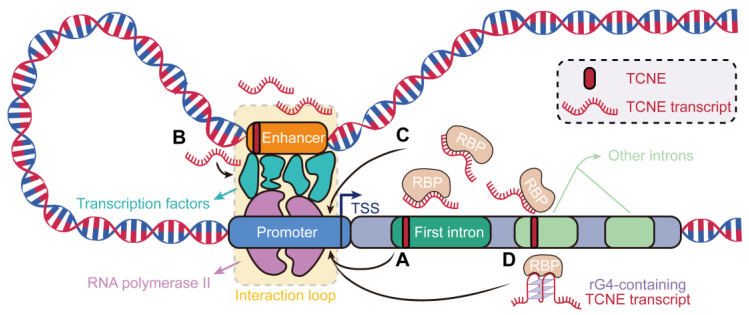
Overview of emerging regulatory and mechanistic paradigms of TCNEs in breast cancer. (**A**) The first intronic TCNEs positively regulate the host genes. (**B**) The transcripts of TCNEs with putative enhancer features promote the formation of enhancer-promoter looping. (**C**) TCNE transcripts recruit RBPs to influence transcription machinery and promote gene expression. (**D**) rG4-containing TCNE transcripts tend to recruit more RBPs and be more stable, contributing to the modulation of gene expression and cellular processes. RBP: RNA-binding protein; rG4: RNA G-quadruplex; and TSS: transcription start site.

Genomic regulatory elements are integral to the regulation of gene expression, influencing chromatin accessibility and modification, as well as TSS activity [56]. Therefore, studying the regulatory mechanisms of TCNEs could provide novel insight into how TCNEs regulate host genes in breast cancer. Our study has discovered that the TCNEs identified in breast cancer samples were predominantly in introns and physically biased towards TSSs of their host genes. Then, we revealed that the first intronic TCNEs could be essential transcription factor binding sites, which were associated with positive regulation of transcriptional progress, and the distances between the intronic TCNEs and the TSSs of the host genes were negatively correlated with the expression levels of the host genes, suggesting that these TCNEs could potentially impact the transcription levels of host genes (Figure 6A). Consistent with earlier perspectives, genomic regulatory elements located within the first intron of host genes can facilitate efficient transcription and boost the overall expression level of host genes [33,57].

TCNEs harbored considerable potential in the intricate landscape of the cancer genome and could function as enhancers to drive the initiation and progression of cancers [14,15]. Here, we found that approximately one-third of TCNEs were supported by known epigenetic and genetic features of active putative enhancers in breast cancer. For example, TRAM2-TCNE, with high enrichment of transcriptional signals, was located in the first intron of TRAM2. TRAM2 is driven by an enhancer to induce epithelial-to-mesenchymal cell transition in breast cancer cells, which is involved in cell proliferation invasion and migration, and high TRAM2 expression is associated with poor survival in cancer patients [35,36]. Additionally, the transcript of TRAM2-TCNE was significantly overexpressed in breast cancer cell lines and was able to potentially perform the eRNA function, promoting the formation of enhancer-promoter looping (Figure 6B). Then, we also discovered that a subset of TCNEs, defined as typical enhancers, played crucial roles in many principal pathways involved in the initiation, invasion, and migration of breast cancer. The expression levels of three TCNEs, defined as risk factors, were statistically significantly higher in breast cancer cell lines than in a normal cell line. It was fair to conjecture that due to a majority of TCNEs being in the vicinity of the gene promoter; the transcripts of these TCNEs could function as eRNAs, facilitating the dynamic interaction between enhancers and the promoters of key genes involved in breast cancer.

Identifying the sequence patterns and structural features in the identified TCNE transcripts facilitates the discovery of various RBP binding motifs incorporated in these regulatory elements, which were crucial for understanding complex regulatory mechanisms. In our study, almost all RBPs, enriched by the transcripts of the identified TCNE, exhibited higher expression levels in breast cancer tissues compared to NAT. Meanwhile, these RBPs were expressed at higher levels in the group with a high expression of TCNEs, indicating a correlation between the expression levels of RBPs and TCNEs. Specifically, the top-ranked RBP motif ENOX1, and the most frequently enriched motif PTBP1, were also shown to be consistent with the overall expression. Experimental evidence has suggested that regulatory elements could highlight the regulatory motifs for RBPs and participate in the control of gene expression to participate in the formation of regulatory networks [58,59]. Here, we further emphasized that the transcripts of the TCNEs could recruit a large number of RBPs that serve to recognize the core promoter to form complexes [39], rendering the complexes more readily available for binding to the promoters of the crucial genes, thereby regulating the expression levels of these genes in breast cancer initiation and development (Figure 6C). Moreover, GO enrichment analysis indicated that these RBPs were significantly relevant to splicing processes and the identified TCNEs could recruit these RBPs to influence the assembly of the core splicing machinery in the vicinity of splice sites and regulate the expression patterns of genes [38,60].

Given that RNA linear chains typically fold into secondary structures, we also probed the presence of non-classical secondary structures on the sequence of TCNE transcripts. As expected, the comprehensive motif analysis showed that rG4 motifs were significantly observed in the sequence of TCNE transcripts. Moreover, rG4-containing TCNE transcripts were structurally more stable and could recruit more RBP motifs, underlining their importance in the modulation of gene expression and cellular process [44,61,62]. For example, one RBP, HNRNPL, recruited by rG4-containing TCNE transcripts, was demonstrated to be an rG4-binding protein in the QUADRatlas database, which could regulate gene expression via its impact on transcript stability and contribute to breast cancer metastasis [46]. These TCNE transcripts were active in breast cancer biological networks that were significantly relevant to breast cancer development and estrogen response. For example, these pathways, known as the Notch signaling, Hedgehog signaling, KRAS signaling DN, P53 pathway, Wnt/β-catenin signaling, and Estrogen response early pathways, have been demonstrated to be classical pathways in breast cancer and participate in tumor growth, proliferation, and metastasis, as well as estrogen response regulation. In particular, rG4-mediated translational elongation stalling was found to affect the proteolysis of the human estrogen receptor [63] and estrogen receptor signaling is a key regulator of cell proliferation, differentiation, and survival in hormone-sensitive cancers, including breast cancer [64]. This evidence further demonstrated that rG4-containing TCNE transcripts could influence the potential for breast cancer invasion and metastasis as well as the effectiveness of hormone therapy (Figure 6D).

Although we have discussed the regulatory mechanisms of TCNEs, it is unclear whether these regulatory processes, dominated by TCNEs, are involved in the regulatory networks of breast cancer biology and what roles they may play. Consequently, predicting target genes is crucial for understanding the biological regulatory networks that TCNEs engage in breast cancer [47,48]. Through our straightforward pipeline to identify the links between TCNEs and target genes, 4251 TCNE-to-gene links were found to be significantly and positively correlated. It was found that almost all TCNEs targeted the nearest genes in all TCNE–gene links. One TCNE universally targeted only one gene, whereas in some cases, one gene could be targeted by several TCNEs. As is widely acknowledged, some regulatory elements are typically located near the TSSs of genes and are fundamental in controlling gene expression [65]. The advantage of this mechanism seems to be that it facilitates the precise regulation of gene expression by TCNEs. Just as multiple CNEs can cluster into a genomic regulatory block [17], several TCNEs can potentially shape a similar regulatory block to be effective in breast cancer development. The fact that one gene can be targeted by multiple TCNEs also reflects the complex regulatory networks that modulate gene expression. In addition, functional annotation analysis indicated that the pathways, enriched by these linked genes, are highlighted as being significantly associated with the initiation and progression of breast cancer. Ultimately, we also verified the reliability of the TCNE-to-gene links based on published Hi-C data on chromatin interactions associated with breast cancer.

Regulatory elements with variants can alter gene expression patterns, contributing to the diversity of biological processes and diseases observed in populations. [52]. In our study, we found that most variants located in TCNEs were intronic SNPs and the genes targeted by variant-containing TCNEs were involved in transcriptional and post-transcriptional processes, consistent with the evidence that *cis*-regulatory elements with variants were associated with transcriptional and post-transcriptional deregulation of gene expression in cancers [66,67]. Furthermore, the carcinogenic pathways involved by these variant-related genes confirmed that variant-containing TCNEs are strongly associated with breast cancer risk. For example, β-catenin is the key effector responsible for the transduction of transducing signals in the canonical Wnt cascade and it triggers the transcription of Wnt-specific genes that are essential for controlling cell fate decisions in many cells and tissues associated with cancer [68,69]. Additionally, we uncovered that expression levels of twenty-one genes significantly correlate with breast cancer-related variants based on eQTL analysis. Specially, LARS expression is suppressed during mammary cell transformation in human breast cancer, leading to impaired leucine codon-dependent translation of growth suppressive genes [53]. Finally, we pointed out that the mutational signatures of these TCNEs were strongly associated with the regulatory machinery in breast cancer. Thus, it was evident that these key genes regulated by the variant-containing TCNEs could enhance tumor formation and proliferation and were pivotal components of the regulatory mechanisms of TCNEs in breast cancer.

Although we conducted bioinformatics analyses as comprehensively as possible, further experiments are still needed in the future to validate our conclusions. For example, the absence of the complete CNE dataset and tissue-specific CAGE-seq profiles may affect prediction accuracy, necessitating molecular biology experiments to functionally characterize candidate regulatory elements. In addition, the lack of clinical information related to breast cancer transcriptomic data led to an incomplete analysis of the clinical characteristics of TCNEs with epigenetic and genetic features. Moreover, the SEU-BRCA cohort was exclusively composed of Chinese breast cancer patients and lacked extensive samples from other countries or regions for further supplementation. The small number of samples and the lack of control adjacent samples were also among the factors limiting the analysis in this study.

## 5. Conclusions

In summary, we established a flexible pipeline to identify TCNEs and validated the robustness and accuracy of the identification pipeline. Our study investigated the TCNE landscape in breast cancer as well as uncovering the epigenetic features, sequence and structure signatures, and clinical relevance of the TCNEs. We also revealed the functional pathways of predicted genes targeted by TCNEs and the association of variant-containing TCNEs with breast cancer progression. Therefore, our study highlighted the emerging functional and mechanistic paradigms of TCNEs in breast cancer, which provided genome-wide insights into the initiation and progression of breast cancer and promoted novel avenues for therapeutic intervention.

## Data Availability

The RNA-seq data from SEU-BRCA cohort are available at the National Genomics Data Center (NGDC, https://ngdc.cncb.ac.cn/bioproject/, accessed on 29 July 2021) with BioProject Accession: PRJCA005965 and GSA for Human Accession: HRA001100. The TCNE identification pipeline, named captureTCNE, comprises a comprehensive manual and a straightforward demo, available via GitHub (https://github.com/weylz/captureTCNE) (accessed on 6 March 2024).

## Supplementary Materials

The following supporting information can be downloaded at https://www.mdpi.com/article/10.3390/biom15050627/s1. Figure S1. Preprocessing processes for public data. (A) Distribution of the length of rG4 in the G4Altas database; (B,C) Gene expression data for TCGA and GTEx before and after removal of batch effects. Figure S2. Profiles of the identified TCNEs in breast cancer. (A) Comparison of H3K27ac and ATAC signal between first intronic TCNEs and other intronic TCNEs; (B) Expression correlation between all first intronic TCNEs and the corresponding host genes; (C–I) The significant strong associations between the first intronic TCNEs and the host genes, where *p* < 2.2 × 10^−16^ and ρ > 0.7; The correlation between (J) the normalized H3K27ac signal, or (K) the normalized ATAC signal and the distance from TCNEs to host gene TSSs; (L) Transcription levels of TCNEs significantly differ in a published dataset (PRJNA739366) including 14 paired breast cancer (BRCA) samples and normal tissues adjacent to the tumors (NAT). Columns refer to TCNEs and rows stand for BRCA (pink) and NAT (green). Figure S3. Survival analysis of the identified TCNEs with known putative enhancer features. (A–J) Kaplan-Meier survival plots show the prognostic relevance of ten TCNEs. Figure S4. Consensus motif analysis of TCNEs in breast cancer. (A) Distribution of TCNE transcripts enriched with the corresponding quantity of RBP motifs; (B) Distribution of pqsfinder scores for rG4-containing TCNE transcripts. Figure S5. Analysis of TCNEs associated genes and variants contained in the TCNEs. (A) Manhattan plot for pathway enrichment analysis of linked genes; (B) GO enrichment analysis of linked genes; (C) Pl3K-Art signaling pathway region of TCNE-associated genes; (D) Distribution of the number of variants covered per TCNEs in breast cancer; Bar plot for (E) variant type, and (F) variant classification of variants located in TCNEs; (G) mutational signatures associated with variants on TCNEs; (H) visualization of the weights assigned to each signature (top), matrix of the trinucleotide contexts for the tumor sample (middle), and the tumor matrix is multiplied by the assigned weights (bottom). Table S1. Primer sequences used for qPCR. Table S2. The results of RNA-binding motif enrichment analysis. Table S3. The results of enrichment analysis for target genes of TCNEs using hallmark gene sets and KEGG, Reactome and WikiPathways of Canonical pathways from curated gene sets. Table S4. The results of enrichment analysis for target genes of TCNEs using GO Biological Process from Enrichr.

## Abbreviations

The following abbreviations are used in this manuscript:

TCNE	Transcribed conserved non-coding element
TSS	Transcription start site
NAT	Normal tissues adjacent to the tumor
RBP	RNA-binding protein
SNP	Single nucleotide polymorphism
CNA	Copy number aberration
MCC	Maximal clique centrality
eQTL	Expression quantitative trait loci
eRNA	Enhancer RNA
rG4	RNA G-quadruplex
