# Supplementary material for: Dissecting the Emerging Regulatory and Mechanistic Paradigms of Transcribed Conserved Non-Coding Elements in Breast Cancer
Source: Biomolecules. 2025 Apr 27;15(5):627. doi: 10.3390/biom15050627 (PMC12108834; doi:10.3390/biom15050627)
Supplement: Supplementary file 1 [file biomolecules-15-00627-s001.zip › Supplementary Figures.pdf]

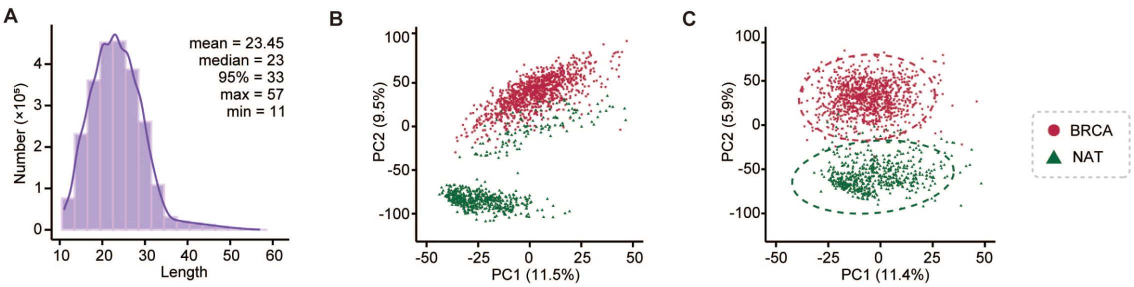

Supplementary Figure S1. Preprocessing processes for public data. (A) Distribution of the length of rG4 in the G4Altas database; (B-C) Gene expression data for TCGA and GTEx before and after removal of batch effects.

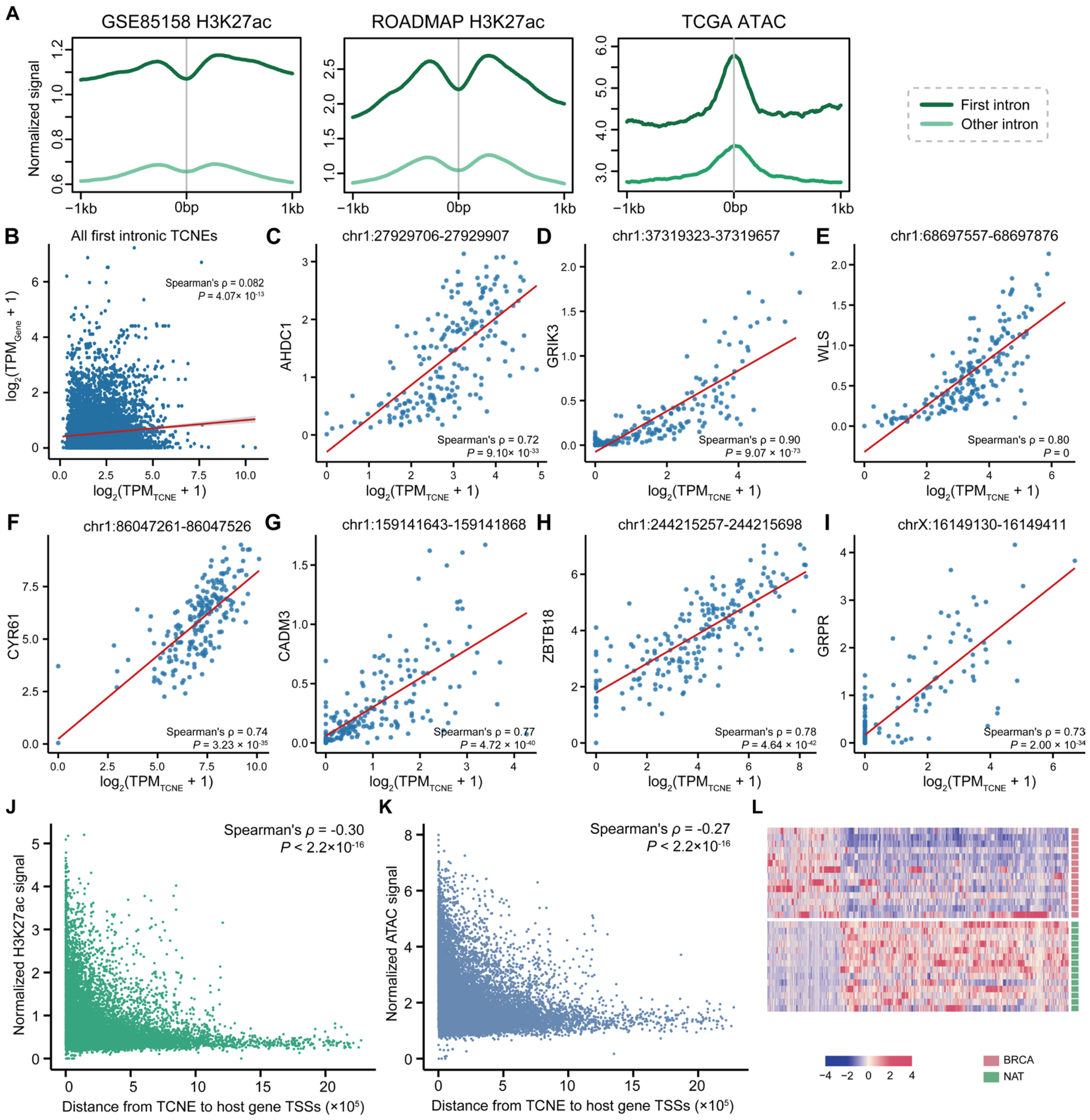

Supplementary Figure S2. Profiles of the identified TCNEs in breast cancer. (A) Comparison of H3K27ac and ATAC signal between first intronic TCNEs and other intronic TCNEs; (B) Expression correlation between all first intronic TCNEs and the corresponding host genes; (C-I) The significant strong associations between the first intronic TCNEs and the host genes, where $P < 2.2 \times 10^{-16}$ and $\rho > 0.7$; The correlation between (J) the normalized H3K27ac signal, or (K) the normalized ATAC signal and the distance from TCNEs to host gene TSSs; (L) Transcription levels of TCNEs significantly differ in a published dataset (PRJNA739366) including 14 paired breast cancer (BRCA) samples and normal tissues adjacent to the tumors (NAT). Columns refer to TCNEs and rows stand for BRCA (pink) and NAT (green).

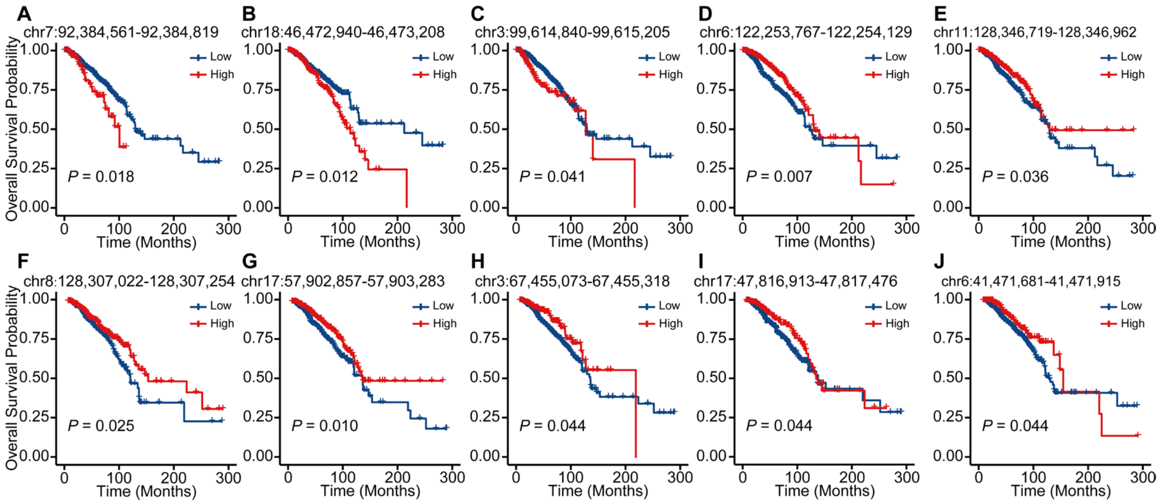

Supplementary Figure S3. Survival analysis of the identified TCNEs with known putative enhancer features. (A-J) Kaplan-Meier survival plots show the prognostic relevance of ten TCNEs.

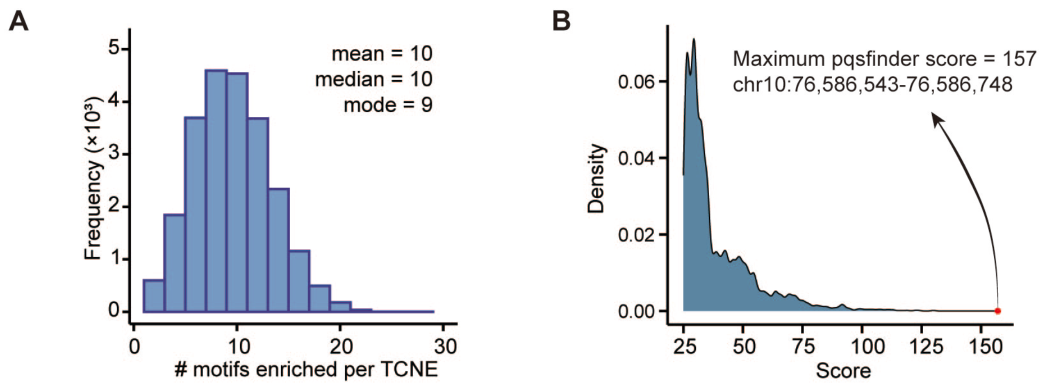

Supplementary Figure S4. Consensus motif analysis of TCNEs in breast cancer. (A) Distribution of TCNE transcripts enriched with the corresponding quantity of RBP motifs; (B) Distribution of pqsfinder scores for rG4-containing TCNE transcripts.

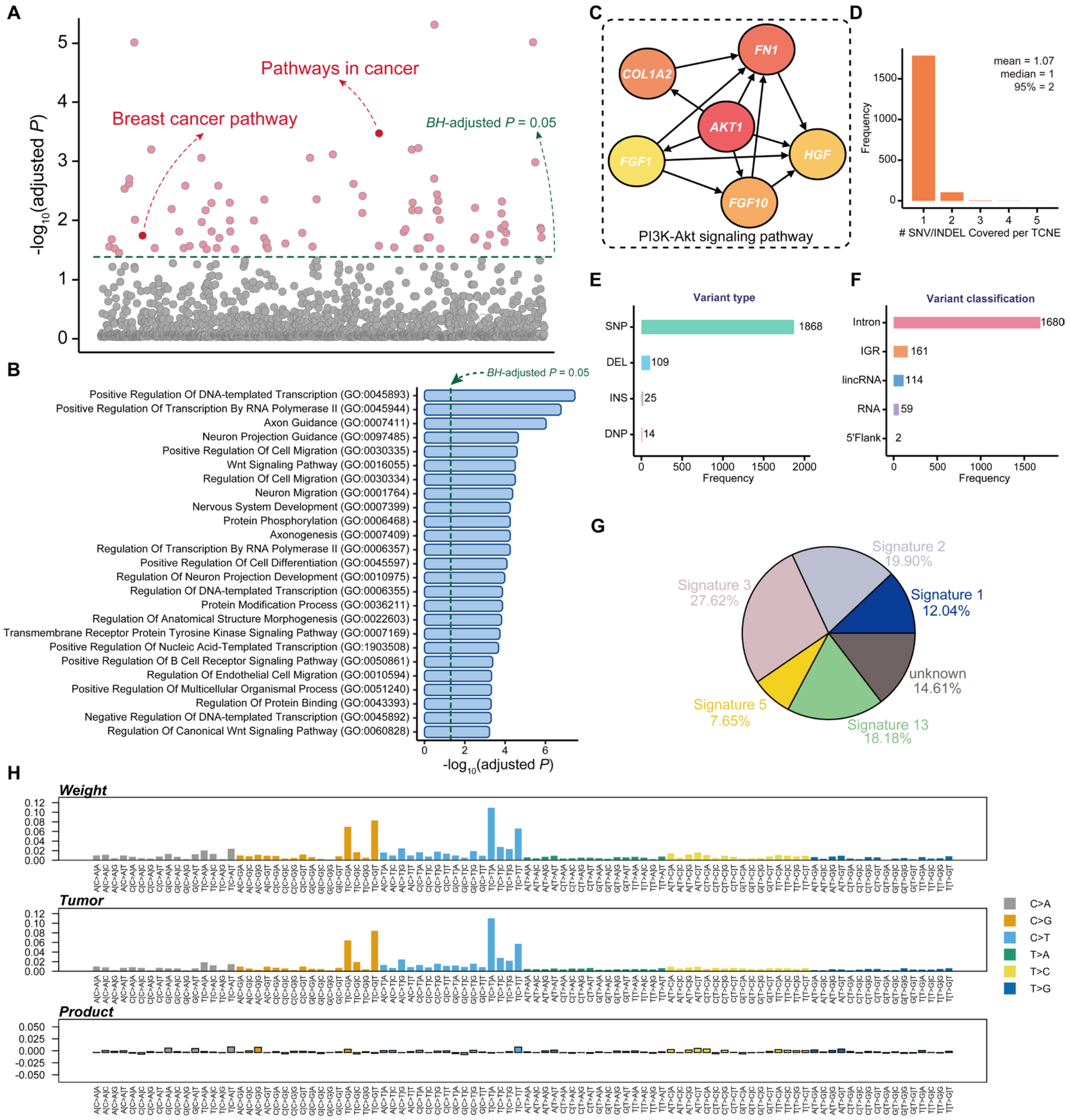

Supplementary Figure S5. Analysis of TCNEs associated genes and variants contained in the TCNEs. (A) Manhattan plot for pathway enrichment analysis of linked genes; (B) GO enrichment analysis of linked genes; (C) PI3K-Art signaling pathway region of TCNE-associated genes; (D) Distribution of the number of variants covered per TCNEs in breast cancer; Bar plot for (E) variant type, and (F) variant classification of variants located in TCNEs; (G) mutational signatures associated with variants on TCNEs; (H) visualization of the weights assigned to each signature (top), matrix of the trinucleotide contexts for the tumor sample (middle), and the tumor matrix is multiplied by the assigned weights (bottom).
