# Supplementary material for: Dissecting the Emerging Regulatory and Mechanistic Paradigms of Transcribed Conserved Non-Coding Elements in Breast Cancer
Source: Biomolecules. 2025 Apr 27;15(5):627. doi: 10.3390/biom15050627 (PMC12108834; doi:10.3390/biom15050627)
Supplement: Supplementary file 1 [file biomolecules-15-00627-s001.zip › Supplementary Tables.pdf]

Supplementary Table S1. Primer sequences used for qPCR.

Name		Primer sequence（5`-3`）
Human-GAPDH	sense	ATTTGGTCGTATTGGGCG
	antisense	CTCGCTCCTGGAAGATGG
chr6:52,416,350-52,416,570	sense	TCTACCTTTTCCTACAGCTGAGT
	antisense	TACTGACCCCTGCAGACCC
chr7:92,384,561-92384,819	sense	TTAACCTCGGGGAACGTCTG
	antisense	TCATGTGAGGTCTTGGCTGG
chr18:46,472,940-46,473,208	sense	ACCAGCTGTCTTAGTTTCCGA
	antisense	CAGTCATTCTGCCAGGCCA
chr3:99614,840-99,615,205	sense	AGGGAACTGCCACGGTTC
	antisense	TCCCTTGCCTTCCTTTTGCA

Supplementary Table S2. The results of RNA-binding motif enrichment analysis.

Rank	Motif ID	RNA-binding Protein	*P* value	Adjust *P* value	E value	PWM min	Target Sequences with Motif	% of Target Sequences with Motif
1	RNCMPT00149	ENOX1	1.33E-125	1.89E-121	1.93E-119	1.02	8,294	35.80
2	RNCMPT00156	CNOT4	1.11E-123	1.43E-119	1.46E-117	1.02	7,562	32.64
3	RNCMPT00089	SRSF10	1.06E-103	2.09E-99	2.13E-97	1.67	7,675	33.13
4	RNCMPT00019	SRSF10	1.23E-92	2.25E-88	2.30E-86	1.02	10,203	44.04
5	RNCMPT00088	SRSF10	2.92E-75	6.50E-71	6.63E-69	1.80	8,139	35.13
6	RNCMPT00001	A1CF	5.79E-72	8.38E-68	8.55E-66	1.10	7,804	33.68
7	RNCMPT00169	KHDRBS1	5.25E-71	8.23E-67	8.39E-65	1.02	8,723	37.65
8	RNCMPT00090	SRSF10	9.40E-71	1.25E-66	1.27E-64	1.02	7,485	32.31
9	RNCMPT00166	BRUNOL5	2.78E-64	4.30E-60	4.38E-58	3.02	3,326	14.36
10	RNCMPT00036	LIN28A	2.13E-60	2.85E-56	2.90E-54	1.11	6,970	30.08
11	RNCMPT00004	BRUNOL4	4.64E-55	8.71E-51	8.89E-49	3.34	2,848	12.29
12	RNCMPT00072	SRSF2	2.59E-51	4.34E-47	4.43E-45	2.15	5,678	24.51
13	RNCMPT00185	KHDRBS2	5.06E-51	8.38E-47	8.54E-45	1.20	7,564	32.65
14	RNCMPT00044	PCBP2	1.83E-50	3.53E-46	3.60E-44	2.97	5,041	21.76
15	RNCMPT00163	SRSF1	1.14E-49	2.05E-45	2.09E-43	1.10	9,387	40.52
16	RNCMPT00108	SRSF1	2.39E-47	2.47E-43	2.52E-41	8.67	2,447	10.56
17	RNCMPT00160	HNRNPH2	5.34E-47	7.46E-43	7.61E-41	4.77	3,130	13.51
18	RNCMPT00109	SRSF1	8.26E-46	8.53E-42	8.70E-40	3.75	3,387	14.62
19	RNCMPT00178	HNRPLL	2.85E-44	3.99E-40	4.07E-38	2.09	4,529	19.55
20	RNCMPT00162	LIN28A	1.03E-43	1.45E-39	1.48E-37	1.04	7,596	32.79
21	RNCMPT00268	PTBP1	1.23E-43	3.10E-39	3.17E-37	1.44	10,677	46.08
22	RNCMPT00055	RBM5	1.35E-41	2.48E-37	2.53E-35	1.71	6,482	27.98
23	RNCMPT00027	HNRNPL	1.68E-41	2.66E-37	2.71E-35	1.84	4,742	20.47
24	RNCMPT00032	HuR	3.74E-37	7.49E-33	7.64E-31	2.66	5,599	24.17
25	RNCMPT00107	SRSF1	1.74E-34	2.25E-30	2.29E-28	2.32	4,435	19.14
26	RNCMPT00063	SAMD4A	1.63E-32	2.29E-28	2.33E-26	1.03	7,577	32.70
27	RNCMPT00187	BRUNOL6	2.31E-32	3.62E-28	3.69E-26	1.08	8,030	34.66
28	RNCMPT00034	KHDRBS3	4.09E-31	4.58E-27	4.67E-25	1.04	6,069	26.19
29	RNCMPT00106	SRSF1	3.70E-31	5.34E-27	5.44E-25	2.65	4,253	18.36
30	RNCMPT00269	PTBP1	2.56E-29	6.08E-25	6.20E-23	2.23	5,063	21.85
31	RNCMPT00171	PABPC5	4.67E-29	1.07E-24	1.09E-22	2.33	5,162	22.28
32	RNCMPT00067	SRSF9	7.38E-29	1.45E-24	1.48E-22	4.67	2,533	10.93
33	RNCMPT00154	RBM5	8.49E-28	1.55E-23	1.58E-21	1.20	8,445	36.45
34	RNCMPT00172	IGF2BP3	2.27E-25	5.56E-21	5.68E-19	2.48	2,702	11.66
35	RNCMPT00086	ZC3H14	9.48E-24	2.08E-19	2.12E-17	1.31	9,738	42.03
36	RNCMPT00184	RBM24	6.18E-22	8.22E-18	8.38E-16	2.62	4,180	18.04
37	RNCMPT00161	FXR1	1.69E-21	1.25E-17	1.28E-15	1.02	4,077	17.60
38	RNCMPT00091	HNRNPL	1.19E-21	1.36E-17	1.39E-15	1.02	6,175	26.65
39	RNCMPT00033	IGF2BP2	1.20E-21	2.92E-17	2.97E-15	2.38	3,955	17.07
40	RNCMPT00150	ESRP2	2.31E-19	4.16E-15	4.24E-13	1.28	8,314	35.88
41	RNCMPT00153	PABPC3	4.13E-19	6.54E-15	6.67E-13	6.12	1,338	5.77
42	RNCMPT00112	HuR	3.79E-17	7.41E-13	7.56E-11	3.61	2,722	11.75
43	RNCMPT00085	ZC3H10	3.16E-16	1.78E-12	1.82E-10	1.03	3,087	13.32
44	RNCMPT00020	FXR2	2.59E-16	2.46E-12	2.50E-10	1.10	4,773	20.60
45	RNCMPT00079	U2AF2	1.05E-14	1.92E-10	1.96E-08	1.15	9,060	39.10
46	RNCMPT00110	SRSF1	2.21E-12	3.32E-08	3.39E-06	1.12	7,371	31.81
47	RNCMPT00053	RBM41	3.99E-12	4.83E-08	4.93E-06	2.21	3,382	14.60
48	RNCMPT00117	HuR	9.15E-12	2.00E-07	2.04E-05	4.49	1,820	7.86
49	RNCMPT00155	PABPC1	5.68E-11	1.22E-06	1.24E-04	16.00	107	0.46
50	RNCMPT00064	SART3	7.79E-11	1.67E-06	1.70E-04	15.60	115	0.50
51	RNCMPT00157	PABPN1	1.38E-09	2.73E-05	2.78E-03	1.31	8,574	37.01
52	RNCMPT00016	FMR1	2.91E-09	3.22E-05	3.28E-03	2.34	2,823	12.18

53	RNCMPT00043	PABPC4	7.71E-09	1.46E-04	1.49E-02	33.30	50	0.22
54	RNCMPT00052	RBM4	1.03E-07	2.17E-04	2.22E-02	2.31	748	3.23
55	RNCMPT00186	PCBP1	3.56E-08	4.73E-04	4.82E-02	9.76	577	2.49

Supplementary Table S3. The results of enrichment analysis for target genes of TCNEs using hallmark gene sets and KEGG, Reactome and WikiPathways of Canonical pathways from curated gene sets.

Rank	Term	Ratio	Adjust *P*	Library	PMID
1	RHO GTPase cycle	59/786	6.44E-06	REACTOME	27216196; 32772212
2	UV Response DN	28/786	1.28E-05	HALLMARK	✗
3	Axon guidance	26/786	1.29E-05	KEGG	36333352
4	Pathways in cancer	42/786	4.42E-04	KEGG	34135756
5	Regulation of actin cytoskeleton	31/786	7.84E-04	KEGG	30707556
6	CDC42 GTPase cycle	25/786	8.30E-04	REACTOME	27216196; 29858187
7	RAC1 GTPase cycle	28/786	8.30E-04	REACTOME	27216196; 29858187
8	Mitotic spindle	29/786	1.01E-03	HALLMARK	29017056
9	ECM proteoglycans	16/786	1.15E-03	REACTOME	33330449
10	Malignant pleural mesothelioma	50/786	1.15E-03	WP	✗
11	VEGFA-VEGFR2 signaling pathway	49/786	1.37E-03	WP	29774034
12	Rett Syndrome Causing Genes	12/786	2.58E-03	WP	✗
13	Arrhythmogenic right ventricular cardiomyopathy (ARVC)	15/786	2.61E-03	KEGG	✗
14	Arrhythmogenic right ventricular cardiomyopathy	15/786	3.16E-03	WP	✗
15	Non-integrin membrane-ECM interactions	13/786	3.16E-03	REACTOME	33383846
16	Development of ureteric collection system	13/786	3.40E-03	WP	✗
17	Extracellular matrix organization	36/786	3.40E-03	REACTOME	34011405
18	Apical junction	27/786	3.83E-03	HALLMARK	30728783
19	RHOA GTPase cycle	22/786	4.76E-03	REACTOME	32772212
20	Nuclear receptors	10/786	5.05E-03	WP	28087820
21	Melanogenesis	17/786	5.76E-03	KEGG	✗
22	Hedgehog signaling pathway	12/786	5.84E-03	KEGG	23547970
23	RHOC GTPase cycle	14/786	6.16E-03	REACTOME	32772212
24	TGF-β signaling pathway	20/786	6.28E-03	WP	32303620
25	Focal adhesion	26/786	6.57E-03	KEGG	29415725
26	RAC3 GTPase cycle	16/786	6.57E-03	REACTOME	29061650
27	Epithelial-mesenchymal transition	26/786	6.67E-03	HALLMARK	29510731
28	Signaling by VEGF	17/786	8.02E-03	REACTOME	29774034

29	CREB1 phosphorylation through NMDA receptor-mediated activation of RAS signaling	8/786	8.43E-03	REACTOME	✗
30	Embryonic stem cell pluripotency pathways	18/786	8.86E-03	WP	32133359
31	RAC2 GTPase cycle	15/786	8.86E-03	REACTOME	28807941
32	Regulation of actin cytoskeleton	21/786	8.86E-03	WP	30707556
33	RHO GTPases Activate WASPs and WAVEs	9/786	8.86E-03	REACTOME	32772212
34	RHOB GTPase cycle	13/786	9.02E-03	REACTOME	32772212
35	Nuclear Receptor transcription pathway	11/786	9.83E-03	REACTOME	28087820
36	TGF-β receptor signaling in skeletal dysplasias	12/786	9.99E-03	WP	✗
37	Basal cell carcinoma	11/786	1.28E-02	KEGG	✗
38	RHOJ GTPase cycle	11/786	1.28E-02	REACTOME	32772212
39	Focal adhesion	25/786	1.32E-02	WP	29415725
40	Glycosaminoglycan metabolism	18/786	1.32E-02	REACTOME	16985046
41	ECM-receptor interaction	14/786	1.40E-02	KEGG	33330449
42	Thyroid hormones production and peripheral downstream signaling effects	15/786	1.56E-02	WP	36004343
43	Wnt signaling	17/786	1.75E-02	WP	33234169
44	TGF-β receptor signaling	11/786	1.79E-02	WP	32303620
45	Protein-protein interactions at synapses	14/786	1.85E-02	REACTOME	34591612
46	Wnt signaling pathway	20/786	1.86E-02	KEGG	33234169
47	Thermogenesis	16/786	1.88E-02	WP	32647572
48	Deactivation of the β-catenin transactivating complex	9/786	2.02E-02	REACTOME	12712206
49	Epithelial to mesenchymal transition in colorectal cancer	21/786	2.02E-02	WP	✗
50	Focal adhesion: PI3K-Akt-mTOR-signaling pathway	33/786	2.02E-02	WP	29415725
51	Prostate cancer	14/786	2.06E-02	KEGG	✗
52	L1CAM interactions	17/786	2.13E-02	REACTOME	32429448
53	Tight junction	18/786	2.13E-02	KEGG	30728783
54	Syndecan interactions	7/786	2.27E-02	REACTOME	36980680
55	Breast cancer pathway	20/786	2.37E-02	WP	31620367
56	Regulation of TP53 Activity through Association with Co-factors	5/786	2.54E-02	REACTOME	34737209
57	Repression of WNT target genes	5/786	2.54E-02	REACTOME	33234169
58	Signaling by WNT	34/786	2.54E-02	REACTOME	33234169

59	Wnt signaling pathway	10/786	2.54E-02	WP	33234169
60	Factors involved in megakaryocyte development and platelet production	21/786	2.60E-02	REACTOME	✗
61	Hedgehog signaling	8/786	2.60E-02	HALLMARK	23547970
62	Adherens junction	12/786	2.74E-02	KEGG	33163938
63	Signaling by TGF-β family members	15/786	2.75E-02	REACTOME	32303620
64	Thyroid cancer	7/786	3.04E-02	KEGG	✗
65	Heparan sulfate/heparin (HS-GAG) metabolism	10/786	3.07E-02	REACTOME	34858858
66	Integrin cell surface interactions	13/786	3.23E-02	REACTOME	33383846
67	EGF/EGFR Signaling Pathway	20/786	3.37E-02	WP	34206026
68	Pathways affected in adenoid cystic carcinoma	11/786	3.52E-02	WP	✗
69	SUMOylation of intracellular receptors	7/786	3.52E-02	REACTOME	33968766
70	Heme metabolism	23/786	3.55E-02	HALLMARK	32010627
71	Diseases of signal transduction by growth factor receptors and second messengers	41/786	3.56E-02	REACTOME	33292604
72	Acute myeloid leukemia	10/786	3.63E-02	KEGG	✗
73	MAPK family signaling cascades	33/786	3.63E-02	REACTOME	19818165
74	Pluripotent stem cell differentiation pathway	9/786	3.75E-02	WP	32493501
75	Cell migration and invasion through p75NTR	7/786	3.85E-02	WP	31966528
76	Deregulation of Rab and Rab effector genes in bladder cancer	5/786	3.85E-02	WP	✗
77	Ethanol effects on histone modifications	7/786	3.85E-02	WP	28899409
78	AKT phosphorylates targets in the nucleus	4/786	3.91E-02	REACTOME	26030190
79	Hippo signaling regulation pathways	14/786	3.91E-02	WP	36347846
80	Regulation of commissural axon pathfinding by SLIT and ROBO	4/786	3.91E-02	REACTOME	✗
81	Phosphorylation of L1	9/786	3.96E-02	REACTOME	✗
82	RHOU GTPase cycle	8/786	3.96E-02	REACTOME	32772212
83	Fragile X syndrome	16/786	4.02E-02	WP	✗
84	Endoderm differentiation	18/786	4.02E-02	WP	33308219
85	Amplification and expansion of oncogenic pathways as metastatic traits	5/786	4.64E-02	WP	37370117

Supplementary Table S4. The results of enrichment analysis for target genes of TCNEs using GO Biological Process from Enrichr.

Rank	Term	Ratio	Adjust *P*	Library
1	Positive Regulation Of DNA-templated Transcription (GO:0045893)	122/1243	3.45E-08	GO Biological Process
2	Positive Regulation Of Transcription By RNA Polymerase II (GO:0045944)	97/938	1.69E-07	GO Biological Process
3	Axon Guidance (GO:0007411)	29/149	9.25E-07	GO Biological Process
4	Neuron Projection Guidance (GO:0097485)	24/124	2.23E-05	GO Biological Process
5	Positive Regulation Of Cell Migration (GO:0030335)	38/272	2.52E-05	GO Biological Process
6	Wnt Signaling Pathway (GO:0016055)	18/76	3.14E-05	GO Biological Process
7	Regulation Of Cell Migration (GO:0030334)	51/434	3.14E-05	GO Biological Process
8	Neuron Migration (GO:0001764)	15/55	4.21E-05	GO Biological Process
9	Nervous System Development (GO:0007399)	50/433	5.57E-05	GO Biological Process
10	Protein Phosphorylation (GO:0006468)	55/500	5.57E-05	GO Biological Process
11	Axonogenesis (GO:0007409)	29/188	5.57E-05	GO Biological Process
12	Regulation Of Transcription By RNA Polymerase II (GO:0006357)	158/2028	5.57E-05	GO Biological Process
13	Positive Regulation Of Cell Differentiation (GO:0045597)	37/283	8.11E-05	GO Biological Process
14	Regulation Of Neuron Projection Development (GO:0010975)	27/174	1.04E-04	GO Biological Process
15	Regulation Of DNA-templated Transcription (GO:0006355)	149/1922	1.35E-04	GO Biological Process
16	Protein Modification Process (GO:0036211)	69/711	1.35E-04	GO Biological Process
17	Regulation Of Anatomical Structure Morphogenesis (GO:0022603)	22/127	1.49E-04	GO Biological Process
18	Transmembrane Receptor Protein Tyrosine Kinase Signaling Pathway (GO:0007169)	36/284	1.79E-04	GO Biological Process
19	Positive Regulation Of Nucleic Acid-Templated Transcription (GO:1903508)	57/557	2.11E-04	GO Biological Process
20	Positive Regulation Of B Cell Receptor Signaling Pathway (GO:0050861)	5/6	4.08E-04	GO Biological Process
21	Regulation Of Endothelial Cell Migration (GO:0010594)	17/88	4.69E-04	GO Biological Process
22	Positive Regulation Of Multicellular Organismal Process (GO:0051240)	43/387	4.79E-04	GO Biological Process
23	Regulation Of Protein Binding (GO:0043393)	20/118	4.93E-04	GO Biological Process
24	Negative Regulation Of DNA-templated Transcription (GO:0045892)	88/1025	4.93E-04	GO Biological Process
25	Regulation Of Canonical Wnt Signaling Pathway (GO:0060828)	28/207	5.91E-04	GO Biological Process
26	Cell Surface Receptor Signaling Pathway Involved In Cell-Cell Signaling (GO:1905114)	9/27	7.76E-04	GO Biological Process
27	Positive Regulation Of Ossification (GO:0045778)	11/41	7.76E-04	GO Biological Process
28	Positive Regulation Of Epithelial Cell Migration (GO:0010634)	17/95	1.04E-03	GO Biological Process

29	Heart Development (GO:0007507)	24/171	1.30E-03	GO Biological Process
30	Generation Of Neurons (GO:0048699)	24/172	1.39E-03	GO Biological Process
31	Cell Morphogenesis Involved In Differentiation (GO:0000904)	8/24	2.25E-03	GO Biological Process
32	Canonical Wnt Signaling Pathway (GO:0060070)	13/65	3.07E-03	GO Biological Process
33	Positive Regulation Of Endothelial Cell Migration (GO:0010595)	15/85	3.53E-03	GO Biological Process
34	Negative Regulation Of Axon Regeneration (GO:0048681)	4/5	3.76E-03	GO Biological Process
35	Negative Regulation Of Nucleic Acid-Templated Transcription (GO:1903507)	45/456	3.76E-03	GO Biological Process
36	Peptidyl-Tyrosine Phosphorylation (GO:0018108)	13/67	3.80E-03	GO Biological Process
37	Positive Regulation Of Cell Motility (GO:2000147)	27/221	3.80E-03	GO Biological Process
38	Ventricular Septum Morphogenesis (GO:0060412)	8/27	4.82E-03	GO Biological Process
39	Regulation Of Synapse Assembly (GO:0051963)	11/51	5.00E-03	GO Biological Process
40	Positive Regulation Of miRNA Transcription (GO:1902895)	10/43	5.36E-03	GO Biological Process
41	Actin Filament Organization (GO:0007015)	20/144	5.60E-03	GO Biological Process
42	Regulation Of Neurotransmitter Transport (GO:0051588)	7/21	5.60E-03	GO Biological Process
43	Positive Regulation Of Osteoblast Differentiation (GO:0045669)	10/44	5.95E-03	GO Biological Process
44	Modulation Of Chemical Synaptic Transmission (GO:0050804)	18/123	5.95E-03	GO Biological Process
45	Phosphorylation (GO:0016310)	42/429	5.95E-03	GO Biological Process
46	Mesonephros Development (GO:0001823)	5/10	5.95E-03	GO Biological Process
47	Mitochondrial Protein Processing (GO:0034982)	5/10	5.95E-03	GO Biological Process
48	Regulation Of Axon Regeneration (GO:0048679)	5/10	5.95E-03	GO Biological Process
49	Negative Regulation Of Transcription By RNA Polymerase II (GO:0000122)	65/763	5.95E-03	GO Biological Process
50	Peptidyl-Tyrosine Modification (GO:0018212)	11/54	6.70E-03	GO Biological Process
51	Ventricular Septum Development (GO:0003281)	9/37	6.70E-03	GO Biological Process
52	Positive Regulation Of Developmental Process (GO:0051094)	27/233	6.70E-03	GO Biological Process
53	Positive Regulation Of Protein Metabolic Process (GO:0051247)	24/196	6.70E-03	GO Biological Process
54	Axon Extension Involved In Axon Guidance (GO:0048846)	4/6	6.70E-03	GO Biological Process
55	Negative Regulation Of Neuron Projection Regeneration (GO:0070571)	4/6	6.70E-03	GO Biological Process
56	Neuron Projection Extension Involved In Neuron Projection Guidance (GO:1902284)	4/6	6.70E-03	GO Biological Process
57	Negative Regulation Of Axon Extension (GO:0030517)	8/30	7.36E-03	GO Biological Process
58	Positive Regulation Of Bone Mineralization (GO:0030501)	9/38	7.43E-03	GO Biological Process

59	Cell-Cell Signaling By Wnt (GO:0198738)	7/23	7.66E-03	GO Biological Process
60	Regulation Of Osteoblast Differentiation (GO:0045667)	14/85	7.67E-03	GO Biological Process
61	Ganglion Development (GO:0061548)	5/11	8.13E-03	GO Biological Process
62	Retinoic Acid Receptor Signaling Pathway (GO:0048384)	5/11	8.13E-03	GO Biological Process
63	Positive Regulation Of Lymphocyte Differentiation (GO:0045621)	9/39	8.43E-03	GO Biological Process
64	Negative Chemotaxis (GO:0050919)	8/31	8.43E-03	GO Biological Process
65	Regulation Of Translation (GO:0006417)	24/202	8.80E-03	GO Biological Process
66	Positive Regulation Of Endothelial Cell Proliferation (GO:0001938)	13/77	9.31E-03	GO Biological Process
67	Positive Regulation Of Wnt Signaling Pathway (GO:0030177)	16/109	9.50E-03	GO Biological Process
68	Regulation Of Bone Mineralization (GO:0030500)	11/58	9.73E-03	GO Biological Process
69	Circulatory System Development (GO:0072359)	18/132	9.73E-03	GO Biological Process
70	Positive Regulation Of miRNA Metabolic Process (GO:2000630)	10/49	9.83E-03	GO Biological Process
71	Regulation Of Cell Adhesion (GO:0030155)	19/144	9.83E-03	GO Biological Process
72	Positive Regulation Of Protein Binding (GO:0032092)	11/59	1.07E-02	GO Biological Process
73	Regulation Of miRNA Transcription (GO:1902893)	11/59	1.07E-02	GO Biological Process
74	Negative Regulation Of Cell Projection Organization (GO:0031345)	10/50	1.10E-02	GO Biological Process
75	Regulation Of Vascular Associated Smooth Muscle Cell Migration (GO:1904752)	5/12	1.10E-02	GO Biological Process
76	Peptidyl-Serine Phosphorylation (GO:0018105)	20/158	1.11E-02	GO Biological Process
77	Positive Regulation Of Epithelial Cell Proliferation (GO:0050679)	17/123	1.11E-02	GO Biological Process
78	Hemopoiesis (GO:0030097)	15/101	1.13E-02	GO Biological Process
79	Skeletal System Development (GO:0001501)	19/149	1.38E-02	GO Biological Process
80	Protein-Containing Complex Organization (GO:0043933)	17/126	1.43E-02	GO Biological Process
81	Ephrin Receptor Signaling Pathway (GO:0048013)	9/43	1.44E-02	GO Biological Process
82	Positive Regulation Of Biomineral Tissue Development (GO:0070169)	9/43	1.44E-02	GO Biological Process
83	Endothelial Cell Differentiation (GO:0045446)	5/13	1.55E-02	GO Biological Process
84	Regulation Of Epithelial To Mesenchymal Transition (GO:0010717)	13/83	1.57E-02	GO Biological Process
85	Regulation Of Axon Extension (GO:0030516)	8/35	1.57E-02	GO Biological Process
86	Positive Regulation Of Antigen Receptor-Mediated Signaling Pathway (GO:0050857)	6/20	1.77E-02	GO Biological Process
87	Negative Regulation Of Neuron Projection Development (GO:0010977)	10/54	1.80E-02	GO Biological Process
88	Regulation Of Smooth Muscle Cell Proliferation (GO:0048660)	10/54	1.80E-02	GO Biological Process

89	Sympathetic Ganglion Development (GO:0061549)	4/8	1.81E-02	GO Biological Process
90	Peptidyl-Serine Modification (GO:0018209)	20/166	1.81E-02	GO Biological Process
91	Positive Regulation Of Canonical Wnt Signaling Pathway (GO:0090263)	13/85	1.84E-02	GO Biological Process
92	Cellular Response To Growth Factor Stimulus (GO:0071363)	19/155	1.94E-02	GO Biological Process
93	Regulation Of BMP Signaling Pathway (GO:0030510)	12/75	1.94E-02	GO Biological Process
94	Negative Regulation Of Cell Differentiation (GO:0045596)	23/207	2.17E-02	GO Biological Process
95	Neuron Projection Morphogenesis (GO:0048812)	18/146	2.46E-02	GO Biological Process
96	Regulation Of Neurotransmitter Secretion (GO:0046928)	8/38	2.51E-02	GO Biological Process
97	Cytoskeleton Organization (GO:0007010)	15/111	2.55E-02	GO Biological Process
98	Regulation Of Cell Motility (GO:2000145)	16/123	2.60E-02	GO Biological Process
99	Cytoskeleton-Dependent Intracellular Transport (GO:0030705)	6/22	2.67E-02	GO Biological Process
100	Dendritic Spine Morphogenesis (GO:0060997)	5/15	2.67E-02	GO Biological Process
101	Sympathetic Nervous System Development (GO:0048485)	5/15	2.67E-02	GO Biological Process
102	Negative Regulation Of Cellular Process (GO:0048523)	46/537	2.67E-02	GO Biological Process
103	B Cell Homeostasis (GO:0001782)	4/9	2.67E-02	GO Biological Process
104	Lung Epithelium Development (GO:0060428)	4/9	2.67E-02	GO Biological Process
105	Protein Autophosphorylation (GO:0046777)	19/162	2.96E-02	GO Biological Process
106	Positive Regulation Of Nervous System Development (GO:0051962)	9/49	3.02E-02	GO Biological Process
107	Regulation Of Actin Cytoskeleton Organization (GO:0032956)	13/91	3.02E-02	GO Biological Process
108	Regulation Of Cytoskeleton Organization (GO:0051493)	15/114	3.02E-02	GO Biological Process
109	Chordate Embryonic Development (GO:0043009)	10/59	3.02E-02	GO Biological Process
110	Negative Regulation Of Developmental Growth (GO:0048640)	7/31	3.05E-02	GO Biological Process
111	Regulation Of Cell Junction Assembly (GO:1901888)	6/23	3.12E-02	GO Biological Process
112	Regulation Of Intracellular Signal Transduction (GO:1902531)	29/297	3.20E-02	GO Biological Process
113	Response To Retinoic Acid (GO:0032526)	10/60	3.32E-02	GO Biological Process
114	Regulation Of B Cell Receptor Signaling Pathway (GO:0050855)	5/16	3.32E-02	GO Biological Process
115	Regulation Of NMDA Receptor Activity (GO:2000310)	5/16	3.32E-02	GO Biological Process
116	Regulation Of Endothelial Cell Proliferation (GO:0001936)	13/93	3.45E-02	GO Biological Process
117	Neuron Projection Development (GO:0031175)	21/192	3.63E-02	GO Biological Process
118	Negative Regulation Of Ion Transmembrane Transporter Activity (GO:0032413)	6/24	3.66E-02	GO Biological Process

119	Outflow Tract Septum Morphogenesis (GO:0003148)	6/24	3.66E-02	GO Biological Process
120	Heparan Sulfate Proteoglycan Biosynthetic Process, Enzymatic Modification (GO:0015015)	4/10	3.66E-02	GO Biological Process
121	Negative Regulation Of Sodium Ion Transmembrane Transporter Activity (GO:2000650)	4/10	3.66E-02	GO Biological Process
122	Negative Regulation Of Cell Adhesion (GO:0007162)	11/72	3.69E-02	GO Biological Process
123	Cellular Component Assembly (GO:0022607)	26/260	3.69E-02	GO Biological Process
124	Positive Regulation Of Peptidyl-Tyrosine Phosphorylation (GO:0050731)	16/130	3.75E-02	GO Biological Process
125	Regulation Of Small GTPase Mediated Signal Transduction (GO:0051056)	15/118	3.75E-02	GO Biological Process
126	Peptidyl-Diphthamide Biosynthetic Process From Peptidyl-Histidine (GO:0017183)	3/5	3.75E-02	GO Biological Process
127	Peptidyl-Diphthamide Metabolic Process (GO:0017182)	3/5	3.75E-02	GO Biological Process
128	Ventricular Compact Myocardium Morphogenesis (GO:0003223)	3/5	3.75E-02	GO Biological Process
129	Positive Regulation Of GTPase Activity (GO:0043547)	24/234	3.75E-02	GO Biological Process
130	Fat Cell Differentiation (GO:0045444)	10/62	3.75E-02	GO Biological Process
131	Epithelial Tube Morphogenesis (GO:0060562)	7/33	3.80E-02	GO Biological Process
132	Sprouting Angiogenesis (GO:0002040)	9/52	3.81E-02	GO Biological Process
133	Activation Of Protein Kinase B Activity (GO:0032148)	6/25	4.12E-02	GO Biological Process
134	Negative Regulation Of Response To Wounding (GO:1903035)	6/25	4.12E-02	GO Biological Process
135	Regulation Of Synapse Organization (GO:0050807)	6/25	4.12E-02	GO Biological Process
136	Semaphorin-Plexin Signaling Pathway (GO:0071526)	7/34	4.40E-02	GO Biological Process
137	Regulation Of Cold-Induced Thermogenesis (GO:0120161)	17/146	4.44E-02	GO Biological Process
138	Positive Regulation Of Cell Population Proliferation (GO:0008284)	41/483	4.44E-02	GO Biological Process
139	Exocytosis (GO:0006887)	12/86	4.44E-02	GO Biological Process
140	Positive Regulation Of T Cell Differentiation (GO:0045582)	10/64	4.44E-02	GO Biological Process
141	Regulation Of Secretion By Cell (GO:1903530)	10/64	4.44E-02	GO Biological Process
142	Regulation Of Stress Fiber Assembly (GO:0051492)	11/75	4.45E-02	GO Biological Process
143	Positive Regulation Of Cold-Induced Thermogenesis (GO:0120162)	13/98	4.45E-02	GO Biological Process
144	Negative Regulation Of Signal Transduction (GO:0009968)	26/267	4.45E-02	GO Biological Process
145	Neural Crest Cell Development (GO:0014032)	8/44	4.45E-02	GO Biological Process
146	ERBB Signaling Pathway (GO:0038127)	9/54	4.45E-02	GO Biological Process
147	Mesenchymal Cell Differentiation (GO:0048762)	9/54	4.45E-02	GO Biological Process
148	Negative Regulation Of Protein Ubiquitination (GO:0031397)	9/54	4.45E-02	GO Biological Process

149	Positive Regulation Of Behavior (GO:0048520)	4/11	4.45E-02	GO Biological Process
150	Smooth Muscle Cell Differentiation (GO:0051145)	4/11	4.45E-02	GO Biological Process
151	Regulation Of Defense Response To Virus (GO:0050688)	5/18	4.49E-02	GO Biological Process
152	Dendritic Spine Organization (GO:0097061)	6/26	4.49E-02	GO Biological Process
153	Regulation Of Cardiac Muscle Cell Contraction (GO:0086004)	6/26	4.49E-02	GO Biological Process
154	Regulation Of Myeloid Cell Differentiation (GO:0045637)	6/26	4.49E-02	GO Biological Process
155	Regulation Of Cell-Matrix Adhesion (GO:0001952)	10/65	4.55E-02	GO Biological Process
156	Dendrite Morphogenesis (GO:0048813)	7/35	4.58E-02	GO Biological Process
157	Central Nervous System Development (GO:0007417)	27/283	4.68E-02	GO Biological Process
